# SNX4 promotes glioblastoma progression through the TGFβ1/Smad signaling pathway and represents a potential therapeutic target

**DOI:** 10.1186/s12967-026-08784-w

**Published:** 2026-08-11

**Authors:** Zhipeng Yao, Pengcheng Xu, Tianxiao Shen, Yunpeng Hua, Shaodong Ding, Yan Xu, Wenhua Zhong, Xinya Zhao, Shan Jiang, Min Qi, Xiaochun Jiang, Shizhang Ling

**Affiliations:** 1 The Translational Research Institute for Neurological Disorders, Department of Neurosurgery, the First Affiliated Hospital of Wannan Medical University (Yijishan Hospital of Wannan Medical University), Wuhu, Anhui China; 2 The Institutes of Brain Science, Wannan Medical University, Wuhu, Anhui China; 3 Anhui Province Digital Brain Engineering Research Center, Wannan Medical University, Wuhu, Anhui China; 4 The School of Basic Medical Sciences, Wannan Medical University, Wuhu, Anhui China

**Keywords:** SNX4, Glioblastoma, Drug Screening, TGFβ1/Smad, Targeted therapy

## Abstract

**Background:**

Glioblastoma multiforme (GBM) is a highly malignant primary brain tumor that still lacks highly specific and clinically effective molecular targets for GBM treatment. Sorting Nexin 4 (SNX4) is an important regulatory molecule involved in intracellular membrane trafficking, protein sorting, and receptor recycling on the cell membrane. However, the roles and mechanism of action of SNX4 in GBM remain unclear.

**Methods:**

RT-qPCR and Western blotting methods were used to measure the expression levels of SNX4 in GBM cell lines and glioma tissue samples from GBM patients. The prognostic role of SNX4 in glioma patients was analyzed by bioinformatic analysis. The roles and underlying mechanism of action of SNX4 in the cell proliferation, cell apoptosis, cell cycle progression, cell migration, and cell invasion of GBM cell lines whose SNX4 gene was silenced or the regulatory pathway of SNX4 was pharmacologically inhibited were examined by the corresponding assays. The role of SNX4 in GBM growth was measured in subcutaneous and intracranial orthotopic tumor models in nude mice. Candidate SNX4-binding small-molecule compounds were discovered by virtual drug screening of a library of small molecule compounds and cell-based drug activity screening.

**Results:**

SNX4 was overexpressed in GBM cell lines and glioma tissue samples from GBM patients measured by RT-qPCR and Western blotting and its high expression was associated with poor prognosis in patients with glioma. SNX4 promoted GBM progression by activating the TGFβ1/Smad signaling pathway in in vitro cell-based assays. Nevertheless, the precise molecular mechanism linking SNX4 to TGFβ receptor trafficking remains to be elucidated. SNX4 also promoted glioma growth in subcutaneous and intracranial orthotopic GBM tumor models in nude mice. Two candidate SNX4-binding small-molecule compounds, G149 and G431, were identified through virtual screening and validated by cell-based assay. Both compounds suppressed GBM growth and were associated with reduced SNX4 expression and inhibition of the TGFβ1/Smad signaling pathway in vitro and in vivo.

**Conclusions:**

Our findings suggest that SNX4 functions as an important regulator of GBM progression, may serve as a potential prognostic biomarker for glioma patients, and may represent a potential therapeutic target.

**Supplementary Information:**

The online version contains supplementary material available at https://doi.org/10.1186/s12967-026-08784-w.

## Introduction

Gliomas are the most common primary malignant tumors of the central nervous system (CNS), accounting for 30%-40% of all primary brain tumors and 80% of all malignant brain tumors [1]. Glioblastoma multiforme (GBM) is the main subtype of gliomas, accounting for about 60% of all gliomas [2]. For GBM patients receiving the current standard treatment, the median overall survival (OS) time is 14.6 months after diagnosis, with a 5-year survival rate of only 9.8% [3]. The currently only available chemotherapeutic drug for treating GBM is temozolomide (TMZ), which is efficacious in some patients, but GBM patients easily develop drug resistance and experience significant side effects shortly after TMZ treatment [4]. The great successes of targeted small molecule inhibitors for solid tumors outside CNS have brought hope to GBM patients. However, clinical trials of the EGFR-targeted small molecule inhibitors showed limited efficacy, with no significant improvement in OS time and progression-free survival (PFS) time, primarily due to the blood-brain barrier (BBB) and tumor intrinsic drug resistance [5]. Thus, exploring new intervention targets and developing their targeted drugs that can cross the BBB will provide additional therapeutic options for glioma patients.

Sorting nexins (SNXs) are a family of proteins that are highly evolutionarily conserved from yeast to mammals. To date, 33 members of SNX family have been identified in human and mice genomes and classified into 11 distinct subfamilies [6]. SNXs family is a diverse and crucial group of proteins that orchestrate anterograde endosomal sorting of intracellular proteins, intracellular protein trafficking internalization and recycling of membrane receptors, and signaling [7]. SNXs also partner with other ancient and conserved multiprotein complexes, the retromer and retriever complexes, in the retrograde regulation of cargo retrieval and recycling, preventing cargo degradation [8].

SNX4, as a member of SNXs family, plays a role in maintaining protein homeostasis in the physiological and pathological settings [7]. Studies have shown that SNX4 gene knockout leads to autophagy defects, indicating that SNX4 is a positive regulator of autophagosome assembly and transport in mammalian cells [8]. The in vivo functions of SNX4 in the nervous system have been studied [6, 9]. SNX4 gene knockout did not affect the neuronal survival and activity in mice [9]. SNX4 is involved in neurotransmitter release by negatively regulating synaptic vesicle docking and release at the active zone of the presynaptic membrane [9]. SNX4 interacts with amphiphysins, recruiting the dynamin to the sites of clathrin-mediated endocytosis in the brain [6]. Furthermore, some studies suggest that SNX4 may promote tumor progression [10]. SNX4 is highly expressed in gastric cancer and is significantly negatively correlated with patient prognosis [10]. In clear cell renal cell carcinoma, both mRNA and protein expression levels of SNX4 are significantly lower than in normal tissues, and SNX4 mRNA expression levels are positively correlated with patient prognosis [11]. SNX4 is involved in the promotion of liver cancer progression by stimulating the secretion of SEC22B (Sect. 22 Homolog B, a vesicle trafficking protein) [12]. However, the role and the underlying mechanism of action of SNX4 in gliomas are still unknown. Therefore, it is paramount to elucidate the role and mechanism of SNX4 in gliomas, and SNX4 may even serve as a therapeutic target for targeted therapy of glioma patients.

In this study, first, bioinformatic analyses showed that SNX4 was highly overexpressed in the GBM cell lines and the tissue samples from GBM patients and SNX4 expression level was negatively correlated with patient survival. Second, in vitro functional experiments revealed that SNX4 significantly enhanced the cell proliferation, invasion, and migration of GBM cells, suppressed apoptosis of GBM cells, and promoted the EMT-like process in GBM cells. Third, we demonstrated that SNX4 promoted GBM growth in vivo in nude mouse xenograft experiments. Fourth, the mechanistic study found that SNX4 promoted GBM progression, at least in part, through the TGFβ/Smad signaling pathway in vitro and in vivo. SNX4 knockdown attenuated TGFβ/Smad signaling, whereas pharmacological activation of the pathway partially rescued the inhibitory phenotypes induced by SNX4 depletion. Conversely, inhibition of TGFβ signaling with Galunisertib significantly attenuated the enhanced proliferation, invasion, and pathway activation induced by SNX4 overexpression, supporting a functional dependence of SNX4-mediated malignant phenotypes on TGFβ/Smad signaling. In addition, activation of the TGFβ pathway increased SNX4 expression, whereas inhibition of the TGFβ pathway reduced SNX4 expression, suggesting the presence of a positive feedback regulatory loop between SNX4 and TGFβ/Smad signaling. Fifth, we discovered two candidate SNX4-binding small-molecule compounds, G149 and G431, with satisfactory anti-GBM efficacy by using the computer-aided methods (AlphaFold, virtual drug screening, and molecular docking) and cell-based drug activity methods. Subsequently, in vitro cell-based assays showed that G149 and G431 strongly inhibited the cell proliferation, migration, and invasion of GBM cells, promoted apoptosis of GBM cells, and suppressed the EMT-like process in GBM cells, while significantly reducing SNX4 expression level. In vivo nude mouse xenograft experiments showed that G149 and G431 significantly inhibited the growth of subcutaneous and intracranial orthotopic GBM xenografts. Their pharmacological effects were accompanied by inhibition of the TGFβ/Smad signaling pathway and closely resembled the phenotypes observed following SNX4 knockdown, supporting the possibility that modulation of SNX4 contributes to their anti-GBM activity. Taken together, these findings suggest that SNX4 contributes to GBM progression, may serve as a potential prognostic biomarker for patients with glioma, and may represent a potential therapeutic target.

## Materials and methods

### Ethics approval and consent to participate

The study was approved (WNMC-AWE-2025195) by the Ethics Committee of the First Affiliated Hospital of Wannan Medical University (Yijishan Hospital of Wannan Medical University). The study was conducted in accordance with the Declaration of Helsinki. A written informed consent from each patient was received prior to participation.

### Sex as a variable

Sex was not considered as a biological variable in our study, although we used human glioma tissue samples from both male and female patients. Our study exclusively established xenograft GBM models in male nude mice. It is unknown whether the findings are relevant for female nude mice.

### Cell culture

Human normal astrocyte cell line (HA 1800), human glioblastoma cell lines (U87 MG, U251 MG, and LN229), and human embryonic kidney cell line 293T (HEK 293T) were obtained from the Cell Bank of the Chinese Academy of Sciences (Shanghai, China). All lines were authenticated for their true identities and free from the contamination of mycoplasma before use. All cells were cultured in DMEM supplemented with 10% FBS and incubated in a 37 °C incubator containing 5% CO_2_. Phosphate Buffered Saline (PBS) and 0.25% Trypsin-EDTA were used for cell passaging.

### Collection of clinical glioma tissue samples

In this study, we collected the glioma tissue and the adjacent tissue samples from glioma patients operated on at the First Affiliated Hospital of Wannan Medical University (Yijishan Hospital of Wannan Medical University). These tissue samples included the 15 cases of glioma adjacent tissue samples (as a normal control group for gliomas), 15 cases of low-grade gliomas (WHO I/II) tissue samples, and 15 cases of high-grade gliomas (WHO III/IV) tissue samples.

### Bioinformatic analysis

The expression level difference of SNX4 in gliomas and its association with survival prognosis of glioma patients were analyzed using the public glioma datasets. First, we used the Gene Expression Profiling Interactive Analysis tool (GEPIA, http://gepia2.cancer-pku.cn/#index) to analyze expression levels of SNX4 in glioma and its correlation with survival in the glioma datasets (LGG and GBM) of The Cancer Genome Atlas (TCGA) (https://www.cancer.gov/ccg/research/genome-sequencing/tcga) database. Second, the Chinese Glioma Genome Atlas (CGGA) RNA-seq data were obtained from the CGGA website (http://www.cgga.org.cn) were analyzed for the expression levels of SNX4 in different grades of glioma and its correlation with the survival prognosis of patients with different grades of glioma. Third, the Visualization Tools for Brain Tumor Datasets website (GlioVis, https://gliovis.bioinfo.cnio.es) was used to analyze the expression level of SNX4 in different grades of glioma in the TCGA database and the correlation between SNX4 and TGFβ1 or TGFβR2.

### Construction of stable SNX4 knockdown GBM cell lines

GBM cell lines with stable knockdown of SNX4 were obtained by lentiviral transfection and further screening. According to our previous protocol [13], shRNA plasmid (based on the plko.1 vector) or control vector plasmid constructed by double digestion was co-transduced into HEK 293T cells with a helper plasmid to obtain lentiviral supernatant, which was then centrifuged and filtered to obtain the lentivirus that was used to infect the target cells, U87 MG, U251 MG and LN229. After 48 h, stable transfected cell lines (shNC, sh1, sh2) were obtained by adding 2 µg/ml puromycin into the lentivirus-infected cells for two weeks. The shRNA sequences were as follows: sh-SNX4-1: 5′-GATCGACTCTATGGTGTATAT-3′, sh-SNX4-2: 5′- GCTGATATTGAACGCTTCAAA-3′, sh-SNX4-NC: 5′- ACGTGTCACGTCAAGAGATTA-3′.

### Transcriptome sequencing (RNA-Seq) and analysis

The expression and enrichment of the differentially expressed genes in U87 MG cells after SNX4 knockdown and the effect of targeting SNX4 small molecule inhibitors on gene expression and enrichment in U87 MG cells were analyzed by transcriptome sequencing. Cells from the stable SNX4 knockdown U87 MG cell line and control cell line, were inoculated in 6-well cell culture plates at a density of 5 × 10^5^ cells per well. Among the U87 MG cells, SNX4 small molecule inhibitors, G149 and G431, were added and incubated for 24 h. Cells were treated with TRIzol and snap frozen with liquid nitrogen. Samples were sent on dry ice to Biozeron, a contract research organization (CRO) (Shanghai, China) for RNA-Seq and analysis. Briefly, after extracting total RNA from the cell samples, rRNA was removed and mRNA was enriched. The library was constructed and sequenced using the Illumina Truseg™ RNA sample prep Kit method. The expression of differential genes and GO and KEGG enrichment analysis were analyzed following the manufacturer’s protocol.

### Virtual drug screening

The virtual screening of the drug was completed by TargetMol Biotechnology (Shanghai, China). Briefly, the AlphaFold was used to predict the structure of the SNX4 protein and then optimization of the AlphaFold-predicted structure of the SNX4 protein at the bond level was performed through the Protein Preparation Wizard module in the Schrödinger software [14]. The MOE-site finder was used to determine all possible binding pocket locations on the target, SNX4 protein. T001, a known active compound library named by TargetMol, was selected for this virtual screening. The T001 compound library was processed using the LigPrep module of the Schrödinger software to maintain the original chirality of the compounds. The prepared receptor and ligand files were imported, and the small molecules in the T001 compound library were subjected to molecular docking using the SP [Standard Precision Mode] screening mode, and after docking, energy optimization was carried out, and the small molecules with the top 20% of the docking score were retained for the next round of screening at each step. Finally, the top 50 small molecules with the highest affinity were selected for the subsequent cell activity screening.

### Cell-based drug activity screening

Firstly, 50 compounds were tested for cell proliferation inhibition on GBM cells (U87 MG, U251 MG, LN229) using CCK8 assay; all compounds were tested at a final concentration of 20 µM. Cells were inoculated in 96-well cell culture plates at a density of 5 × 10^4^ cells per well except the wells without cells that were used as a blank control, and the wells with cells that were treated with solvent only were used as a vehicle control (VC). 3 replicate wells were set up for all groups. Please refer to the “CCK8 assay” section for the rest of the steps for CCK8 assay. Inhibition % = (1- (OD_Compound_ - OD_Blank_) / (OD_VC_ - OD_Blank_)) × 100%. The compounds with percentages of inhibition greater than 50% were subjected to detection of IC50 values. Then the IC50 values of the compounds to inhibit cell proliferation of GBM cells (U87 MG, U251 MG, LN229) were detected by CCK8 assay. The starting concentration of all compounds tested was 10 µM, and the concentration was serially diluted, and the OD value of the cells was detected after incubation for 24 and 48 h, respectively, and the compounds with smaller IC50 were further validated. The toxicity of the compounds on normal human astrocyte cells, HA 1800, and their effects on cell proliferation, migration, invasion, apoptosis and cell cycle of GBM cells (U87 MG, U251 MG, LN229) were detected by EdU assay, wound healing assay, Transwell assay, apoptosis assay, cell cycle assay, respectively, and the active compounds were selected according to the results for the subsequent animal experiments.

### In vivo drug treatment assay

Intracranial orthotopic tumor-bearing nude mice were randomly divided into three groups (*n* = 5/group): control group, G149 treatment group, and G431 treatment group. The treatment groups were injected with 100 µl of G149 (15 mg/kg) and G431 (15 mg/kg) via tail vein, respectively. The drug treatments were administered every other day. Mice were weighed every other day. The fluorescence intensity of intracranial tumor in nude mice was detected every four days until day 15 using animal live imaging system (IVIS Lumina Series Ⅲ, PerkinElmer, USA). The survival of the mice was also recorded daily, and after the mice were euthanized, the brain tissue and five major organs were removed to make paraffin sections for subsequent experiments.

### Statistical analysis

All data were presented as mean ± SEM (standard error of mean). Statistical analyses were carried out with GraphPad Prism 6.01 (GraphPad Software, USA). The two-tailed Student’s t-test was used to define statistical significance between groups. *p* < 0.05 was considered statistically significant.

## Results

### SNX4 is highly overexpressed in glioblastoma and associated with poor survival and prognosis in glioma patients

**Fig. 1:**
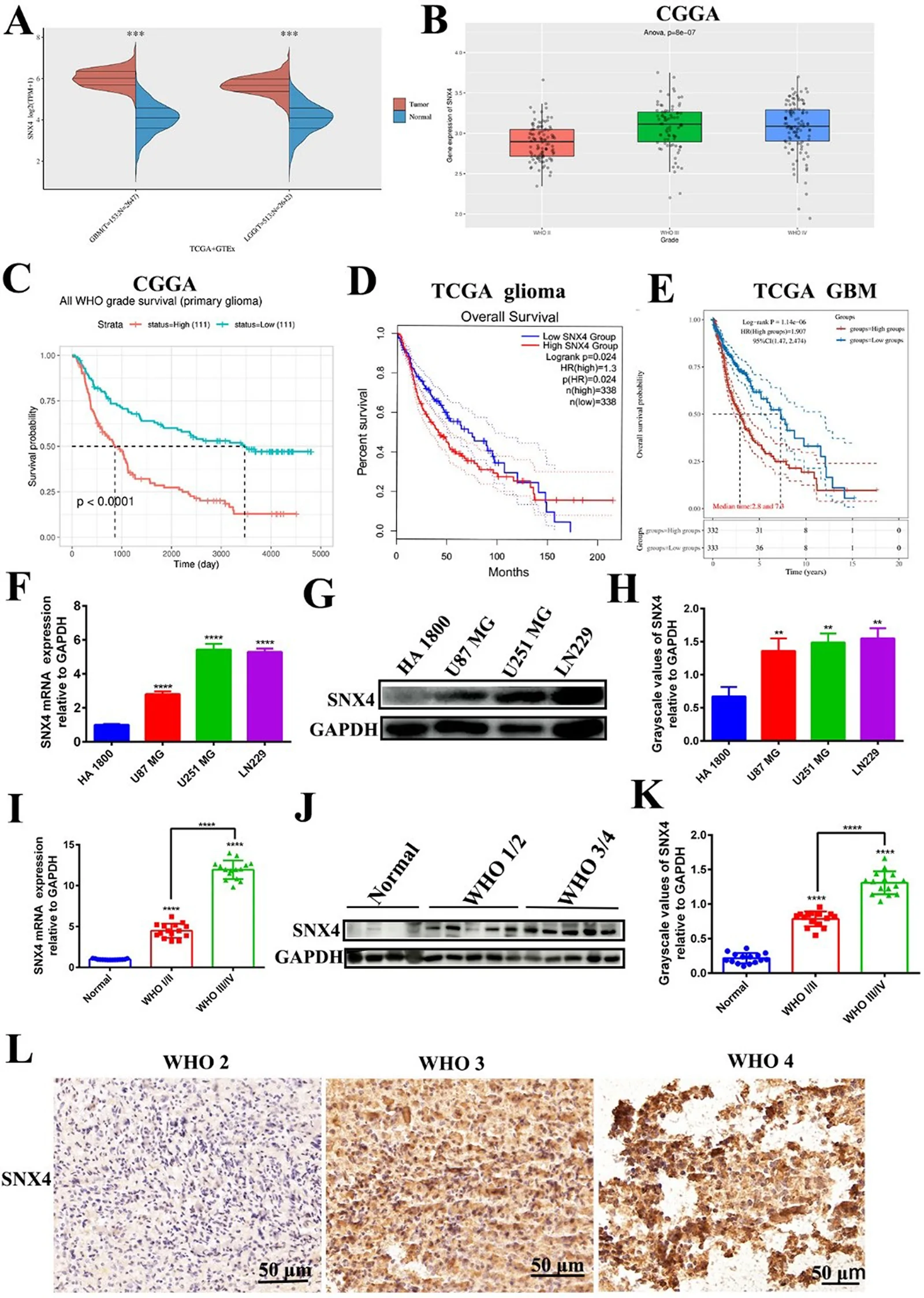
The expression level of SNX4 and the relationship of SNX4 level with patient survival in two public glioma datasets, as well as expression level of SNX4 in GBM cell lines and clinical glioma tissue samples. (**A**) SNX4 expression levels in GBM and LGG compared to normal tissues in TCGA and GTEx glioma datasets. (**B**) The relative expression differences of SNX4 in grade II, III, and IV glioma patients in the CGGA database. (**C**) Analysis of the correlation between SNX4 expression and overall survival in all WHO-grade glioma patients in the CGGA database. (**D**) The correlation between the expression level of SNX4 and the overall survival of GBM and LGG patients in the TCGA database. (**E**) The correlation between SNX4 expression level and overall survival of GBM patients in the TCGA database, showing the significant difference of the two median overall survival times (2.8 vs. 7.3 years). (**F**) Detection of relative expression differences of SNX4 in human normal astrocyte cell line (HA 1800) and three human GBM cell lines by RT-qPCR. (**G**) Detection of relative expression differences of SNX4 in human normal astrocyte cell line and three human GBM cell lines by WB. (**H**) Quantitative analysis and statistical analysis of the grayscale value in the panel G. (**I**) Detection of relative expression differences of SNX4 in normal human brain tissue and glioma tissues of different WHO-grades (I/II vs. III/IV) by RT-qPCR. (**J**) Detection of relative expression differences of SNX4 in normal human brain tissue and glioma tissues of different WHO-grades (I/II vs. III/IV) by WB. (**K**) Quantitative analysis and statistical analysis of the grayscale values in the panel J. (**L**) IHC staining to detect expression differences of SNX4 in clinical glioma tissue samples of different WHO grades (II, III, and IV). All data were expressed as the mean ± SEM of three independent experiments and statistically analyzed by Student’s t-test for P-values. Compared with NC, ** *P* < 0.01; *** *P* < 0.001; **** *P* < 0.0001

First, we analyzed the differences of SNX4 expression between gliomas and the normal groups in the TCGA glioma datasets and GTEx database. The results showed that the expression level of SNX4 was significantly higher than that of the normal group in both LGG and GBM patients (Fig. 1A, Fig S1A). Then we analyzed the expression of SNX4 in the gliomas of different grades in the CGGA and TCGA glioma datasets. The results showed that the higher the grade of glioma, the higher the expression of SNX4, and the expression of SNX4 in patients with grade IV gliomas was significantly higher than that in patients with grade II and grade III gliomas (Fig. 1B, Fig S1B), indicating that the expression level of SNX4 is positively correlated with the malignancy degree of gliomas. In addition, in the TCGA LGG and GBM cohorts, SNX4 expression was significantly higher in IDH‑wild‑type gliomas compared to IDH‑mutant gliomas (Fig S1C). We also examined SNX4 expression in relation to 1p/19q codeletion status. SNX4 expression was significantly elevated in non‑codeleted gliomas compared to 1p/19q‑codeleted gliomas (Fig S1D). Since 1p/19q codeletion is a hallmark of oligodendrogliomas and is associated with better prognosis, whereas 1p/19q-co-deletion rarely occurs in GBM patients, this further supports the role of SNX4 as a biomarker of more aggressive type of gliomas. Using the Verhaak classification (proneural, classical, and mesenchymal), we found that SNX4 expression was also high in the mesenchymal subtype, which is the most aggressive GBM subtype, and lowest in the proneural subtype (Fig S1E). This distribution is particularly notable because the mesenchymal subtype is characterized by activation of the TGFβ pathway, EMT, and inflammatory responses—all of which are functionally linked to SNX4 in our study. The correlation between SNX4 and mesenchymal features further strengthens the mechanistic connection that we have established between SNX4 and the TGFβ1/Smad signaling pathway.

Next, we analyzed the effect of SNX4 expression on the survival of glioma patients in CGGA and TCGA glioma datasets. The survival analyses of glioma patients in the CGGA dataset showed that patients of both all WHO grades (Fig. 1C) and grades II and III (Fig S1F-G) with high SNX4 expression had significantly shorter overall survival (OS) than those with low SNX4 expression. Likewise, the result of the survival analyses in the TCGA glioma patient dataset (Fig. 1D-E) is consistent with the results of the survival analyses in the CGGA dataset.

Finally, we validated SNX4 expression in our own samples of human GBM cell lines and glioma and adjacent normal brain tissue samples surgically resected from glioma human patients. The expression level of SNX4 in 3 GBM cell lines (U87 MG, U251 MG, LN229) was significantly higher than that of human normal astrocyte line, HA 1800, as shown by RT-qPCR (Fig. 1F), WB (Fig. 1G-H), and cytoimmunofluorescence staining (Fig S1H-I). Furthermore, SNX4 expression level in glioma tissues was significantly higher than that of adjacent normal brain tissues, as shown by RT-qPCR (Fig. 1I), WB (Fig. 1J-K), immunofluorescence staining (Fig S1J-K), and immunohistochemical staining (Fig. 1L) and the higher the malignancy of gliomas, the higher the expression level of SNX4, which was consistent with the results of the bioinformatic analysis of TCGA glioma dataset (Fig. 1A) and CGGA dataset (Fig. 1B). In conclusion, SNX4 is highly overexpressed in GBM and significantly associated with poor prognosis and survival of glioma patients.

### SNX4 promotes GBM cell proliferation, migration, invasion and inhibits GBM cell apoptosis

**Fig. 2:**
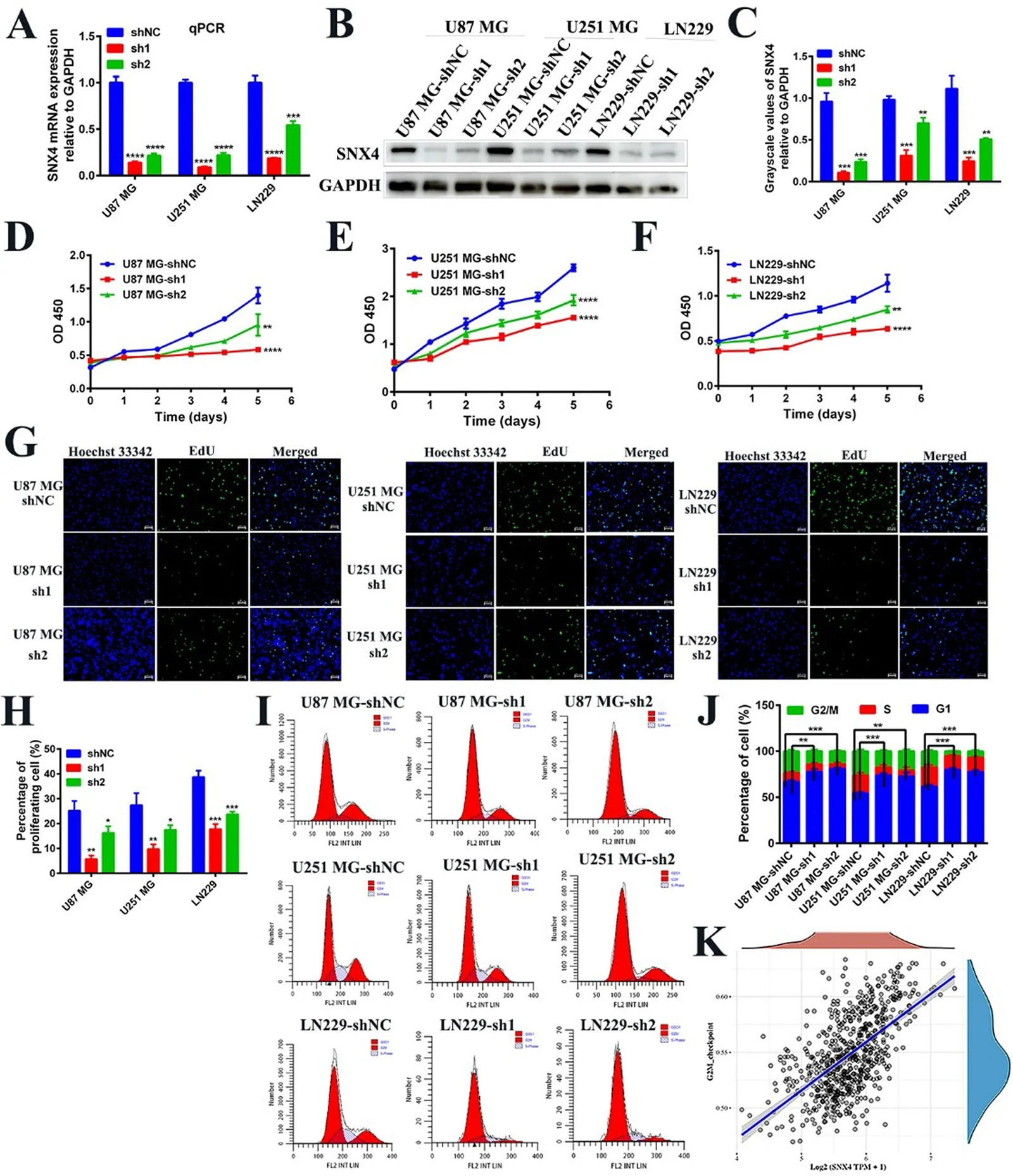
The effect of SNX4 knockdown on the proliferation of GBM cells. (**A**) RT-qPCR shows the knockdown efficiency of shRNA on SNX4 mRNA in GBM cells (U87 MG, U251 MG, LN229). (**B**) WB shows the knockdown effect of shRNA on SNX4 protein in GBM cells (U87 MG, U251 MG, LN229). (**C**) Quantitative and statistical analyses of the grayscale values of protein bands of SNX4 and GAPDH in the panel B. (**D-F**) CCK-8 assay shows the effect of SNX4 knockdown on the proliferation of U87 MG (**D**), U251 MG (E), and LN229 (F) cells. (**G**) EdU assay shows the effect of SNX4 knockdown on the proliferation of GBM cells (U87 MG, U251 MG, LN229). (**H**) Statistical analysis of the percentage of proliferating cells in the panel G. (**I-J**) The representative images of flow cytometry (**I**) and quantitative and statistical analyses (**J**), showing the effect of SNX4 knockdown on the cell cycle of GBM cells (U87 MG, U251 MG, LN229). (**K**) Correlation between the mRNA level of SNX4 gene and G2/M phase was analyzed in TCGA glioma dataset. All data were expressed as the mean ± SEM of three independent experiments and statistically analyzed by Student’s t-test for P-values. Compared with NC, *P < 0.05; ** *P* < 0.01; *** *P* < 0.001; **** *P* < 0.0001

After we successfully established the stable SNX4-knockdown GBM cell lines (U87 MG-sh, U251 MG-sh, LN229-sh), in which the knockdown efficiency of SNX4-sh1 was significantly higher than SNX4-sh2, as shown by RT-qPCR (Fig. 2A) and WB experiments (Fig. 2B-C). SNX4 knockdown significantly inhibited the cell proliferation of the three types of GBM cells in a SNX4 knockdown degree -dependent manner, as shown by CCK8 assay (Fig. 2D-F) and EdU assay (Fig. 2G-H). In addition, flow cytometry showed that SNX4 knockdown inhibited the G2/M phase of GBM cells and significantly arrested the GBM cells in the G1 phase (Fig. 2I-J), which is also consistent with the results of KEGG enrichment in the TCGA glioma dataset (Fig. 2K). Wound healing and Transwell assays demonstrated that SNX4 knockdown significantly inhibited the migratory ability (Fig S2A-C) and invasive ability (Fig S2D-E) of GBM cells in a SNX4 knockdown degree-dependent manner, compared with the control group. Furthermore, we found that SNX4 knockdown promoted apoptosis of GBM cells, as shown by flow cytometry (Fig S2F-I), and increased the expression levels of the pro-apoptotic proteins, cleaved Caspase 3 (C-Caspase 3) and BAX, but decreased the expression level of the major anti-apoptotic protein, BCL-2, in the apoptotic pathway by WB (Fig S2J-K), which also further indicates that SNX4 knockdown promotes apoptosis GBM cells. In summary, SNX4 knockdown inhibits the cell proliferation, migration, invasion of GBM cells and promotes apoptosis of GBM cells.

### SNX4 promotes cell migration, invasion and EMT-like processes in GBM cells through the TGFβ1/Smad signaling pathway

**Fig. 3:**
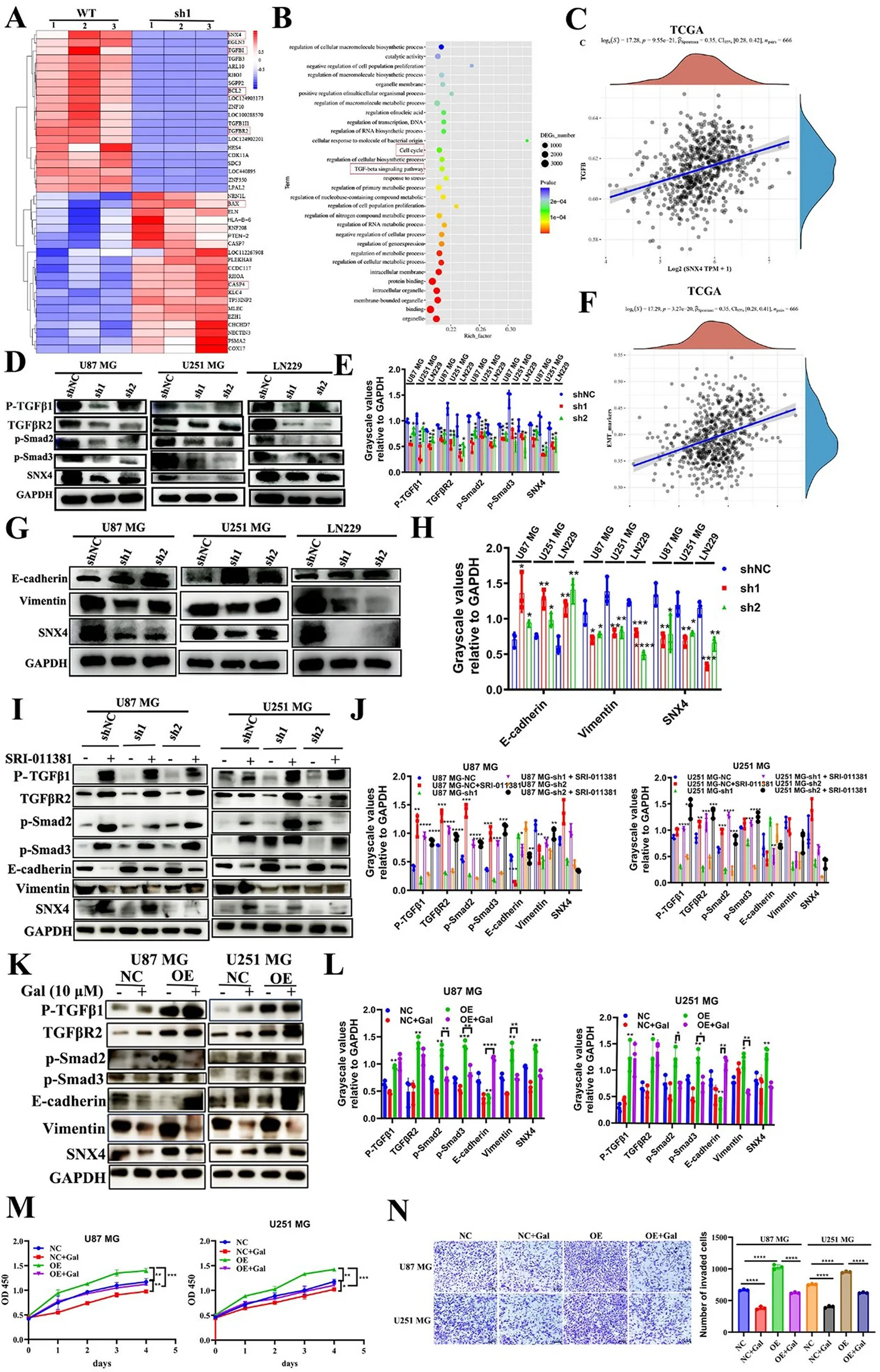
Functional interaction between SNX4 and the TGFβ1/Smad signaling pathway in GBM. (**A**) The gene clustering analysis shows the top 30 differentially expressed genes shown as a heatmap from the RNA-Seq results of U87 MG and U87 MG-sh1 cells. (**B**) GO functional enrichment analysis of the differentially expressed genes (DEGs) from the RNA-Seq results of U87 MG and U87 MG-sh1 cells. (**C**) Analysis of the correlation between the SNX4 mRNA level and the TGFβ pathway in TCGA glioma dataset. (**D-E**) The representative composite images of WB showing the effects of SNX4 knockdown on the expression level and phosphorylation status of the four indicated key component proteins of the TGFβ1/Smad pathway in U87 MG, U251 MG, and LN229 cells from three groups (shNC, sh1, sh2) (D) and the quantitative and statistical analyses of the grayscale values of the protein bands in the panel D (E). (**F**) Analysis of the correlation between the SNX4 mRNA level and EMT markers in TCGA glioma dataset. (**G-H**) The representative composite images of WB showing the effect of SNX4 knockdown on the expression level of the two indicated key proteins involved in the EMT-like process in U87 MG, U251 MG, and LN229 cells from three groups (shNC, sh1, sh2) (G), and the quantitative and statistical analyses of the grayscale values of the protein bands of the EMT markers (H). (**I-J**) The representative composite images of WB showing the effect of SNX4 knockdown on the expression level and phosphorylation status of the four indicated key component proteins of the TGFβ1/Smad pathway and the expression level of the two indicated key proteins involved in the EMT-like process in U87 MG and U251 MG cells treated with TGFβ1 agonist, SRI011381 (I) in three groups (shNC, sh1, sh2), and the quantitative and statistical analyses of the grayscale values of the protein bands in the panel I (J). **(K**–**L**) Representative Western blot images and quantitative analyses showing the effects of Galunisertib on the expression of SNX4, TGFβR2, p-Smad2, and p-Smad3 in SNX4-overexpressing U87 MG and U251 MG cells. (**M**) CCK-8 assays showing that pharmacological inhibition of TGFβ signaling significantly attenuated the enhanced proliferative capacity induced by SNX4 overexpression. (**N**) Transwell invasion assays demonstrating that Galunisertib markedly reduced the increased invasive ability of SNX4-overexpressing GBM cells. All data were expressed as the mean ± SEM of three independent experiments and statistically analyzed by Student’s t-test for P-values. Compared with NC, *P < 0.05; ** *P* < 0.01; *** *P* < 0.001; **** *P* < 0.0001

To explore the molecular mechanism of SNX4 in GBM, we performed transcriptome sequencing of stable SNX4 knockdown GBM cell lines. The heatmap of differential gene analysis showed that among the top 30 of the differentially expressed genes (DEGs), SNX4 was significantly downregulated, confirming that SNX4 gene knockdown was successful (Fig. 3A); BCL-2 was downregulated, whereas BAX and CASP4 (Caspase 4), the pro-apoptotic proteins, were highly upregulated in U87 MG-sh1 cells, compared with the parental control cells (Fig. 3A), which is also consistent with the results of apoptosis and the major component proteins in the apoptotic pathway that were detected by flow cytometry and Western blotting, respectively (Fig S2F-K). TGFβ1 and TGFβR2, the main component genes, in the TGFβ signaling pathway, were also downregulated (Fig. 3A); GO enrichment analysis (Fig. 3B) and KEGG enrichment analysis (Fig S3A-B) indicated that SNX4 was closely related to the TGFβ1/Smad signaling pathway. Furthermore, we found that the expression level of SNX4 was positively correlated with the TGFβ signaling pathway (Fig. 3C) and was strongly positively correlated with TGFβ1 and TGFβR2 (Fig S3C-D) in the TCGA glioma dataset.

We further examined the protein expression levels of four major component proteins of TGFβ1/Smad signaling pathway by WB. We found that TGFβ1 precursor (Pro-TGFβ1, P-TGFβ1), TGFβR2, p-Smad2 and p-Smad3 were significantly downregulated in SNX4 knockdown GBM cell lines (Fig. 3D-E). These results suggest that SNX4 may regulate the proliferation, invasion and migration of GBM cells through the TGFβ1/Smad signaling pathway. We also found that the expression level of EMT markers was significantly positively correlated with SNX4 expression level in the TCGA glioma dataset (Fig. 3F), whereas the knockdown of SNX4 gene significantly increased the protein level of E-cadherin and decreased the protein level of Vimentin in the GBM cell lines (Fig. 3G-H), suggesting that SNX4 is involved in the EMT-like process, and most likely also regulated by the TGFβ1/Smad signaling pathway in GBM cells.

Next, we verified whether the involvement of SNX4 in GBM cell migration, EMT-like processes, and apoptosis was achieved through the TGFβ1/Smad signaling pathway. We treated U87 MG cells (U87 MG-shNC, U87 MG-sh1, U87 MG-sh2) and U251 MG cells (U251-shNC, U251-sh1, U251-sh2) with the TGFβ1 agonist, SRI-011381 (10 µM) for 24 h, then examined the regulatory role of the TGFβ1/Smad signaling pathway on GBM cell migration and EMT-like processes by wound healing and WB. Wound healing experiments showed that the migration ability of U251 MG-shNC, U251 MG-sh1 and U251 MG-sh2 cells was significantly enhanced after SRI-011381 treatment, which almost counteracted the inhibitory effect of SNX4 knockdown on the migration ability of U251 MG (Fig S3E-F). The WB experiments showed that the expression levels of the four major component proteins of the TGFβ1/Smad signaling pathway and SNX4 protein itself were all significantly increased to a similar degree in U87 MG and U251 MG cells (shNC, SNX4-sh1, and SNX4-sh2) after SRI-011381 treatment (Fig. 3I-J, Fig S3G-I), indicating that SNX4 gene knockdown does not disrupt the integrity of TGFβ1/Smad signaling pathway in terms of cellular responsiveness to SRI-011381 and the activation of TGFβ1/Smad signaling pathway upregulates SNX4 expression. Furthermore, we examined whether the activation of TGFβ1/Smad signaling pathway affected the role of SNX4 in the EMT-like process in GBM cells treated with SRI-011381. First, we found that SNX4 gene knockdown increased the expression level of the epithelial marker, E-cadherin, but decreased the expression level of the mesenchymal marker, Vimentin, in two of three GMB cell lines tested (Fig. 3I-J), which is consistent with the results of Fig. 3G, again suggesting that SNX4 promotes EMT-like process in the GBM cell lines. Then we found that SRI-011381 significantly decreased the E-cadherin level, but increased Vimentin level, in U87 MG and U251 MG cells (shNC, SNX4-sh1, and SNX4-sh2) (Fig. 3I-J), suggesting that the promoting role of SNX4 in the EMT-like process in GBM cells can be further potentiated by the TGFβ1/Smad signaling pathway, thus enhancing the invasive ability of the GBM cells. We examined the expression levels of the major component proteins in the apoptotic pathway by WB. The results revealed that compared with control cells (shNC), SNX4 gene knockdown increased the levels of pro-apoptotic proteins (cleaved Caspase 3 and BAX), but decreased the level of anti-apoptotic protein, BCL-2, indicating that SNX4 protects GBM cells from apoptosis (Fig S3J-K). In contrast, compared with control cells (shNC), the expression levels of C-Caspase 3 and BAX was markedly reduced and BCL-2 expression level was elevated in SNX4 gene knockdown GBM cells (sh1 and sh2), after SRI-011381 treatment, indicating that the activation of TGFβ1/Smad signaling pathway promotes apoptosis in SNX4 knockdown cells (Fig S3J-K). This result may be attributed to the insufficient apoptosis-inhibitory effect of SNX4 due to SNX4 gene knockdown or SNX4-independent effect of the activation of TGFβ1/Smad signaling pathway on apoptosis in SNX4 knockdown cells, although the activation of TGFβ1/Smad signaling pathway moderately restored the expression level of SNX4 in SNX4 knockdown cells only in sh1 group. Taken together, the above results suggest that the TGFβ1/Smad signaling pathway is involved in the regulation of SNX4 for GBM cell migration, EMT-like processes, and apoptosis. To further determine whether activation of the TGFβ pathway is required for SNX4-mediated malignant phenotypes, SNX4-overexpressing (SNX4-OE) GBM cells were treated with the TGFβ receptor I inhibitor, Galunisertib (Gal). Western blot analysis demonstrated that SNX4 overexpression markedly increased TGFβR2, p-Smad2 and p-Smad3 levels (Fig. 3K-L), whereas treatment with Galunisertib effectively abolished Smad2/3 phosphorylation without affecting SNX4 expression. CCK-8 assays showed that SNX4 overexpression significantly promoted cell proliferation, whereas Galunisertib largely attenuated this effect (Fig. 3M). Similarly, Transwell assays demonstrated that pharmacological inhibition of TGFβ signaling markedly reduced the increased invasive capacity induced by SNX4 overexpression (Fig. 3N). These findings indicate that activation of the TGFβ pathway is required, at least in part, for SNX4-mediated malignant phenotypes.

### SNX4 promotes GBM tumor growth in mice

**Fig. 4:**
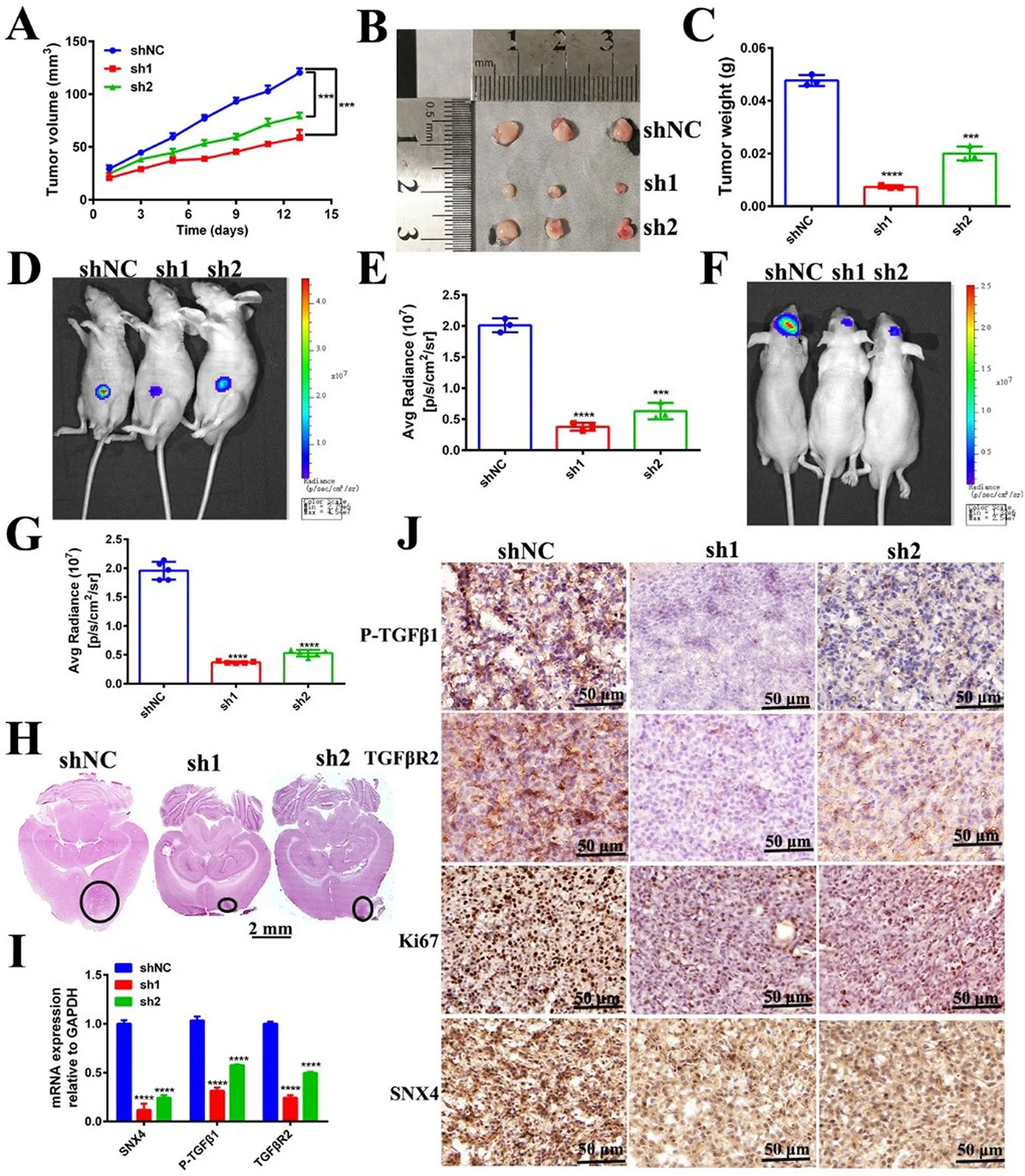
In vivo validation of the effects and mechanisms of action of SNX4 on GBM progression in a subcutaneous and an intracranial orthotopic xenograft GBM tumor model of nude mice. (**A**) The quantitative and statistical analyses of the volumes of subcutaneous tumors measured every 2 days in mice in SNX4 knockdown groups (sh1 and sh2) and control group (shNC) after the subcutaneous injection of U87 MG-Luc cells. (**B**) Images showing the sizes of the excised subcutaneous tumors formed from the subcutaneously injected U87 MG-Luc cells in SNX4 knockdown groups (sh1 and sh2) and control group (shNC) on day 15 (the day of mouse sacrifice) after the subcutaneous injection of U87 MG-Luc cells in mice (*n* = 3 per group). (**C**) The bar graph showing the average tumor weights of the excised of subcutaneous tumors in SNX4 knockdown groups (sh1 and sh2) and control group (shNC) on (the day of mouse sacrifice) and the statistical analysis of tumor weights between the groups. (**D**) The representative bioluminescence images of the subcutaneous tumors formed from the subcutaneously injected U87 MG-Luc cells in mice of SNX4 knockdown groups (sh1 and sh2) and control group (shNC) on day 15 (the day of mouse sacrifice) after the subcutaneous injection of U87 MG-Luc cells in mice (*n* = 3 per group). (**E**) Quantitative analysis of the bioluminescence intensity from the luciferase-luciferin biochemical reaction occurred in subcutaneous tumor tissues in mice in the panel D. (**F**) The representative bioluminescence images of the intracranial tumors formed from the intracranially injected U87 MG-Luc cells in control group (shNC) and SNX4 knockdown groups (sh1 and sh2) mice on day 15 (the day of mouse sacrifice) after the intracranial injection of U87 MG-Luc cells (*n* = 5 per group). (**G**) Quantitative and statistical analyses of the bioluminescence intensity in intracranial tumor tissues from mice in the panel F. (**H**) The representative photomicrographs of the HE staining results of the formalin-fixed paraffin-embedded coronal sections of mouse brain tissue with the intracranial xenograft glioma tissue (encircled in a black oval) in each indicated group of mice, scale bar, 2 mm. (**I**) RT-qPCR detection of the gene expression differences of SNX4, TGFβ1, and TGFβR2 mRNAs in the subcutaneous tumor tissues excised on day 15 in each indicated group of mice, as shown in the panel B. (**J**) The representative photomicrographs of the IHC staining results of the formalin-fixed paraffin-embedded tissue sections of the intracranial glioma tissues excised on day 15, showing the protein expression differences of SNX4, TGFβ1, TGFβR2, and Ki67 in each indicated group of mice. All data were expressed as the mean ± SEM of three independent experiments and statistically analyzed by Student’s t-test for P-values. Compared with NC, *P < 0.05; ** *P* < 0.01; *** *P* < 0.001; **** *P* < 0.0001

Next, we used the subcutaneous xenograft GBM model and the intracranial orthotopic transplant GBM model in nude mice to investigate whether the above in vitro results were also true in vivo. In the subcutaneous xenograft GBM model, compared with the control shNC group, the tumor volumes in the SNX4 knockdown group of mice were significantly smaller and tumor growth rates were slower (Fig. 4A) and the final tumor size and tumor weight measured on the sacrifice day (day 15) were also markedly smaller (Fig. 4B-C); animal live imaging found that compared with the control shNC group, the average fluorescence was significantly weaker (Fig. 4D-E, Fig S4A-B), and the higher the degree of SNX4 knockdown (sh1 vs. sh2), the slower the tumor growth (Fig. 4A-E, Fig S4A-B), suggesting that the tumor volume in the SNX4 knockdown group of mice was significantly smaller. Furthermore, we constructed an intracranial orthotopic transplant GBM model to investigate the effect of SNX4 knockdown on the tumorigenicity, growth, and invasion of U87 MG cells. Animal live imaging results showed that at the first-time imaging on day 5 after the injection of U87 MG-Luc cells, tumors were readily detectable in the shNC group of mice, whereas tumors were barely detectable in the sh1 and sh2 groups of mice, and the intracranial fluorescence range (Fig. 4F, Fig S4A, bottom panel) and the fluorescence intensity (Fig. 4F-G, Fig S4A-B, bottom panel) of tumor in the shNC group of mice were gradually enlarged and increased over time (up to 15 days), respectively, indicating that the GBM tissues were gradually invading into the surrounding brain parenchyma and the number of U87 MG cells in the tumor tissue was significantly greater due to the increased cell proliferation rate (Fig. 4J, Fig S4C-D). In contrast, the in vivo imaging results showed that compared with tumor in the shNC group of mice, the increases of the intracranial fluorescence range (Fig. 4F, Fig S4A, bottom panel) and the fluorescence intensity (Fig. 4F-G, Fig S4A-B, bottom panel) of tumors in the SNX4 knockdown groups (sh1 and sh2) were both significantly slower in the same period of time, suggesting that SNX4 knockdown inhibited the tumorigenicity, cell proliferation and invasion of U87 MG cells in vivo, which were further confirmed by the results of HE staining (Fig. 4H) and Ki67 IHC staining (Fig. 4J, Fig S4C-D). HE staining of brain tissues showed that intracranial GBM tumors of the sh1 and sh2 group mice were significantly smaller than the shNC group mice (Fig. 4H). The results of IHC staining of brain tissues demonstrated that the expression levels of P-TGFβ1, TGFβR2, Ki67, and SNX4 in the tumor tissues of the sh1 and sh2 group mice were significantly lower than the control group mice (Fig. 4J, Fig S4C), suggesting that SNX4 expression level and the cell proliferation rate remained low in vivo in the stable SNX4 knockdown cells. RT-qPCR results of the subcutaneous tumor tissues showed that the expression levels of SNX4, P-TGFβ1 and TGFβR2 in tumor tissues of the sh1 and sh2 group mice were significantly lower than the control group mice (Fig. 4I). The results of IHC staining of the subcutaneous tumor tissues showed that the expression levels of Ki67 and SNX4 were significantly lower in the tumor tissues of the sh1 and sh2 group mice than the control group mice (Fig S4D). Taken together, these results indicate that SNX4 knockdown significantly inhibits the growth of GBM in mice and SNX4 may be a potential therapeutic target for GBM.

### Identification of candidate SNX4-binding small-molecule compounds

**Fig. 5:**
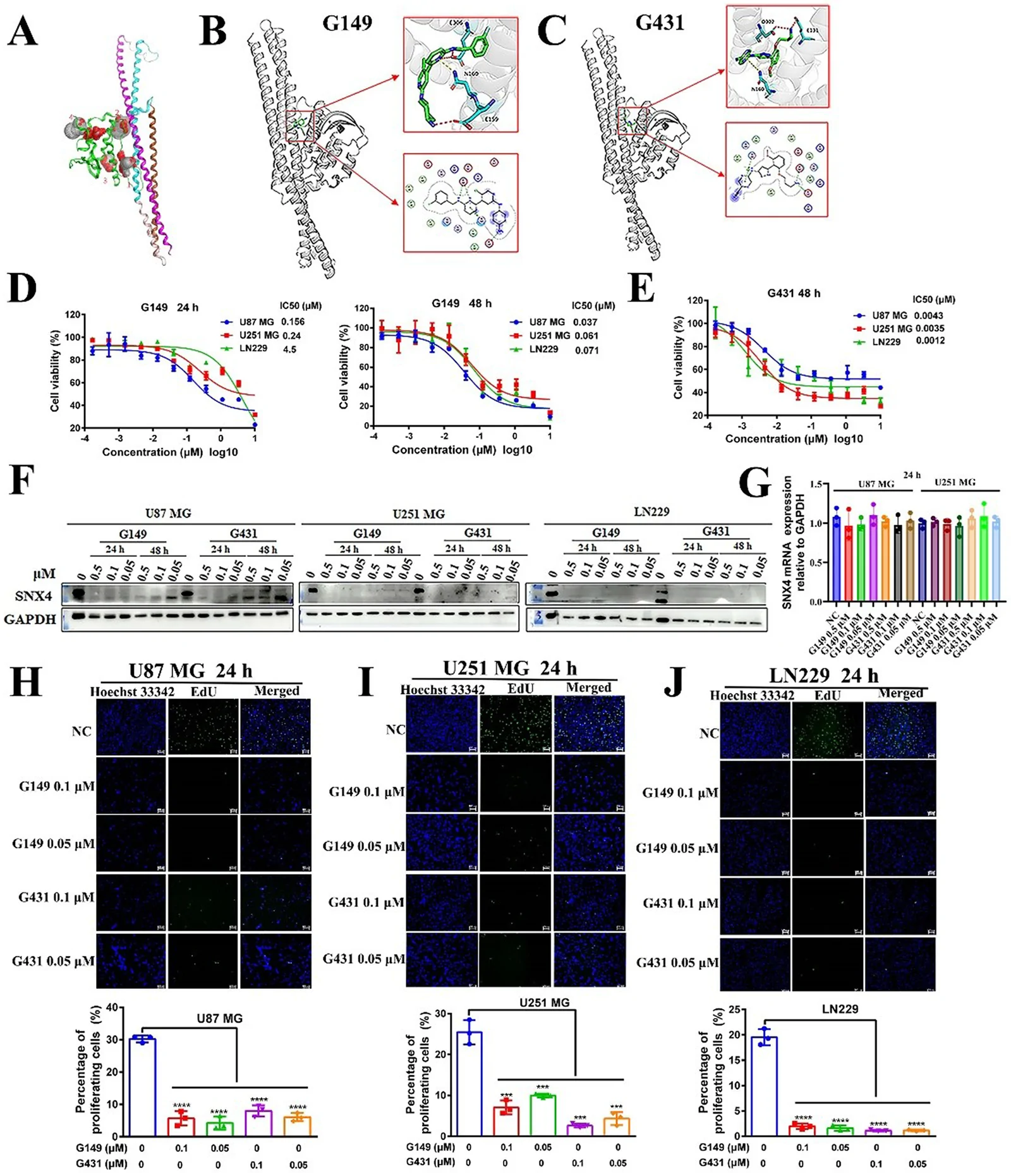
Virtual drug screening of candidate SNX4-binding small-molecule compounds and cell-based assay for drug activity screening. (**A**) The predicted molecular structure of the SNX4 protein by AlphaFold, and the identification of all possible binding pockets in the structure of SNX4 protein by MOE-site finder. The numbers 1–4 denote the four binding pockets. (**B**) The schematic diagram of molecular docking simulation showing the binding of the G149 to the SNX4 protein. The small box denotes the binding site and the two enlarged boxes denote the amino acid residues of SNX4 protein to which G149 binds. (**C**) The schematic diagram of molecular docking simulation showing the binding of the G431 to the SNX4 protein. The small box denotes the binding site, and the two enlarged boxes denote the amino acid residues of SNX4 protein to which G431 binds. (**D**) CCK8 assay-based measurements of the IC50 values of the G149 for inhibiting the cell viability of GBM cell lines (U87 MG, U251 MG, LN229) at 24 h and 48 h of G149 treatment. (**E**) CCK8 assay-based measurements of the IC50 values of the G431 for inhibiting the cell viability of GBM cell lines (U87 MG, U251 MG, LN229) at 48 h of G431 treatment. (**F**) The expression levels of SNX4 protein measured by WB analysis in GBM cell lines (U87 MG, U251 MG, LN229) treated with G149 or G431 at the indicated concentrations (0.05 µM and 0.1 µM) and periods of time (24 h and 48 h). (**G**) The levels of SNX4 mRNA measured by RT-qPCR analysis in GBM cell lines (U87 MG, U251 MG) treated with G149 or G431 at the indicated concentrations (0.05 µM, 0.1 µM and 0.5 µM) and period of time (24 h). (**H**-**J**) The representative images of EdU fluorescence staining showing the effects of G149 and G431 on the proliferation of U87 MG (H), U251 MG (**I**), and LN229 (**J**) cells treated with G149 or G431 at the indicated concentrations (0.05 µM and 0.1 µM) for 24 h, and the quantitative and statistical analyses of the EdU fluorescence staining results (the bottom parts of the corresponding panels **H**-**J**). All data were expressed as the mean ± SEM of three independent experiments and statistically analyzed by Student’s t-test for P-values. Compared with NC, *P < 0.05; ** *P* < 0.01; *** *P* < 0.001; **** *P* < 0.0001

To explore the therapeutic potential of targeting SNX4 in GBM, we identified candidate small molecule inhibitors targeting SNX4 by computer-aided approaches (AlphaFold, virtual drug screening, molecular docking) and cell-based drug activity screening. AlphaFold was used to predict the protein structure of SNX4, and then MOE-site finder was used to identify all the possible drug binding pockets (sites) in SNX4 protein. It turned out that SNX4 has four drug binding pockets (Fig. 5A), among which the site 1 has the largest PLB (Protein Ligand Interaction Fingerprints), indicating that it has a higher possibility of a drug binding site (Fig S5A). The virtual drug screening also revealed more drugs with strong affinity for the site 1, compared with the sites 2–4 (Fig S5B-E), so the site 1 was selected as the drug binding pocket. The top 50 drug compounds with the strongest binding affinities for SNX4 protein were selected for the subsequent cell-based drug activity screening. The drug activity of the top 50 small molecule compounds (20 µM) against GBM cell lines (U87 MG, U251 MG, LN229) was verified by CCK8 assay, and the results showed that the compounds G149 and G431 showed the highest efficacy inhibiting the cell proliferation of the three GBM cell lines (Fig S5F-H). Therefore, G149 and G431 were selected for subsequent molecular docking study. Molecular docking identified the binding sites of SNX4 protein for G149 (Fig. 5B) and G431 (Fig. 5C). The results of molecular docking show the amino group on G149 can form hydrogen bonds with E159 on the SNX4 protein, while the amino groups on the pyridine and adjacent to the pyridine ring interact with E306 through hydrogen bonds; additionally, the pyridine ring of the compound interacts with N160 through an Arene-H bond. Likewise, the amino group on G431 can form hydrogen bonds with D302 and E131 on the SNX4 protein, and the pyrazine ring of the compound interacts with N160 through an Arene-H bond. These molecular docking analyses predict favorable binding poses and potential interactions between SNX4 and G149 or G431. We next used CCK8 assay to find out the IC50 values of G149 and G431 for inhibiting the cell proliferation of GBM cells (U87 MG, U251 MG, LN229). The results showed that G149 treatment effectively inhibited the cell proliferation of GBM cells of the three cell types in a time-dependent manner. For 24 h treatment group, G149 achieved the best inhibitory effect on U87 MG and U251 MG cells, with the IC50 values of less than 0.3 µM (Fig. 5D); For 48 h treatment group, the IC50 value was less than 0.1 µM, and the best inhibitory effect was on U87 MG, with the IC50 value of 0.037 µM (Fig. 5D). Likewise, G431 had a strong inhibitory effect on GBM cells of three types after 48 h treatment, with the IC50 values not greater than 5 nM, among which the best inhibitory effect was on LN229, with the IC50 value of 1.2 nM (Fig. 5E). Based on the IC50 values, 0.1 µM and 0.05 µM of G149 and G431, respectively, were selected to subsequently treat the GBM cells of the three types for 24 h and 48 h, and then the SNX4 protein level was verified by WB. The results showed that G149 and G431 had strong inhibitory effects on SNX4 protein level in three GBM cell lines, even at a low concentration (0.05 µM) for as short as 24 h treatment (Fig. 5F), suggesting that G149 and G431 reduce SNX4 protein abundance in GBM cells, although the underlying molecular mechanism requires further investigation. To determine whether G149 and G431 induced reduction of SNX4 protein was associated with altered transcription, SNX4 mRNA levels were measured by RT-qPCR after treatment with G149 or G431 (0.05, 0.1, and 0.5 µM for 24 h) in U87 MG and U251 MG cells. No significant changes in SNX4 mRNA expression were observed at any concentration in either cell line (Fig. 5G). These results indicate that the reduction in SNX4 protein is not accompanied by decreased SNX4 mRNA expression, suggesting that G149 and G431 may regulate SNX4 level through mechanisms beyond transcriptional regulation. The effects of G149 and G431 on the cell proliferation of the three GBM cell lines were further verified by EdU assay, and the results showed that G149 and G431 significantly inhibited the cell proliferation rates of the three GBM cell lines in a concentration-dependent manner (Fig. 5H-J), which is consistent with the results of the inhibition of cell proliferation by SNX4 knockdown approach reported above in this study.

Next, the toxic effects of G149 and G431 on normal human astrocyte cell line, HA 1800, and the effects of G149 and G431 on apoptosis, migration and invasion of three GBM cell lines were examined. Apoptosis assay revealed that G149 and G431 hardly caused apoptosis in HA 1800, even at a high concentration (0.1 µM) for 48 h (Fig S6A-B). However, G149 and G431 caused apoptosis in a concentration- and time-dependent manner in three GBM cell lines, among which the pro-apoptotic effect of G431 on LN229 was stronger, which is also consistent with the results of IC50 assay (Fig S6A-B). The invasion assay showed that G149 and G431 inhibited GBM cell invasion, even at a low concentration (0.05 µM) for as short as 24 h, in which G431 had a stronger invasion-inhibiting effect on LN229 (Fig S6C-D). Migration assay showed that G149 and G431 hardly affected the migration of HA 1800 (Fig S6E-F), but inhibited the migration of two GBM cell lines (U251 MG and LN229) tested, even at a low concentration (0.05 µM) for as short as 24 h (Fig S6G-J). Cell cycle experiments showed that G149 hardly affected the cell cycle progression of HA 1800, while it arrested the cell cycle of the three GBM cell lines in G1 phase (Fig S7), which is also consistent with the results that SNX4 knockdown inhibited the cell cycle progression reported above in this study. In summary, G149 and G431 show barely a toxic effect to HA1800 cells, while they promote apoptosis and inhibit the migration and invasion of GBM cells. The biological effects of G149 and G431 were largely consistent with those observed following SNX4 knockdown, supporting the possibility that SNX4-binding and inhibition of these two candidate inhibitors may contribute to their anti-GBM activity. However, additional studies are required to determine the specificity of these compounds and to exclude potential off-target effects.

### Fluorescently labeled candidate compounds exhibit brain distribution in mice

The main reason for the poor effect of chemotherapeutic and molecularly targeted therapeutic drugs to GBM is that these drugs cannot sufficiently penetrate the BBB to reach the tumor. To verify whether the inhibitors G149 and G431 can readily cross the BBB, we conjugated G149 and G431 with NBD-Cl, respectively. NBD-Cl is a non-fluorescent agent that reacts with thiol or amino to produce a conjugate with strong green fluorescence. The amino groups in the G149 and G431 molecules react with the Cl group on NBD-Cl to produce fluorescent G149-NBD conjugate (Fig S8A) and G431-NBD conjugate (Fig S9A), respectively. High-resolution mass spectrometry (HRMS) detection found that the molecular weights of G149-NBD conjugate (Fig S8B) and G431-NBD conjugate (Fig S9B) were consistent with the theoretically calculated values, indicating that the synthesis of G149-NBD and G431-NBD conjugates was successful.

First, to investigate whether G149-NBD and G431-NBD conjugates can cross the plasma membrane of GBM cells and bind to SNX4 protein localized in the cytoplasm in GBM cells, we incubated G149-NBD and G431-NBD with three GBM cell lines for 24 h, respectively, and the fluorescence imaging revealed that the green fluorescence of G149-NBD or G431-NBD overlapped well with the red fluorescence of SNX4 in the cytoplasm (Fig S8C, Fig S9C), forming the yellow color, indicating that G149 and G431 can readily cross the plasma membrane, co-localize with SNX4, and most likely bind to SNX4. Next, we examined whether G149 and G431 can cross the BBB in vivo. We injected G149-NBD or G431-NBD (15 mg/kg) via tail vein in nude mice bearing intracranial orthotopic GBM tumor, then detected the drug fluorescence in the whole body of mice at different time points (0.5 h, 2 h, 6 h, 24 h, and 48 h) and in the isolated major organs on 48 h. The drug fluorescence was readily observed in the mouse brain 0.5 h after injection of G149-NBD, and the fluorescence intensity began to slowly decrease after 2 h, but remained strong enough to be detectable up to 24 h after injection (Fig S8D-E), and the fluorescence imaging of the isolated the five major organs showed that the brain was still fluorescently strong enough (Fig S8F). Likewise, stronger drug fluorescence was also observed in the mouse brain 0.5 h after injection of G431-NBD and the drug fluorescence gradually intensified over time, until the fluorescence noticeably weakened at 48 h (Fig S9D-E), and the fluorescence imaging of the isolated five major organs showed that the fluorescence of mouse brain was still the strongest (Fig S9F). To dissect the mechanism of BBB crossing, we performed an additional control experiment in which G149-NBD or G431-NBD were intravenously injected into normal (non-tumor-bearing) mice with an intact BBB. The results revealed that fluorescence was readily detected in the brains of normal mice as early as 0.5 h post-injection, although the signal intensity was quantitatively lower than that in orthotopic tumor-bearing mice (Fig S8G-H, Fig S9G-H). These fluorescence imaging results suggest that the NBD-labeled G431 conjugate exhibited a longer duration of detectable fluorescence signal in the brain than the G149 conjugate. However, these observations should be interpreted cautiously because the fluorescent conjugates may not fully reflect the pharmacokinetic behavior of the parental compounds. Based on these results, G149 or G431 was injected every other day during drug treatment of intracranial orthotopic GBM tumor model in the subsequent experiments.

### Candidate SNX4-binding compounds exhibit anti-GBM activity in vivo

**Fig. 6:**
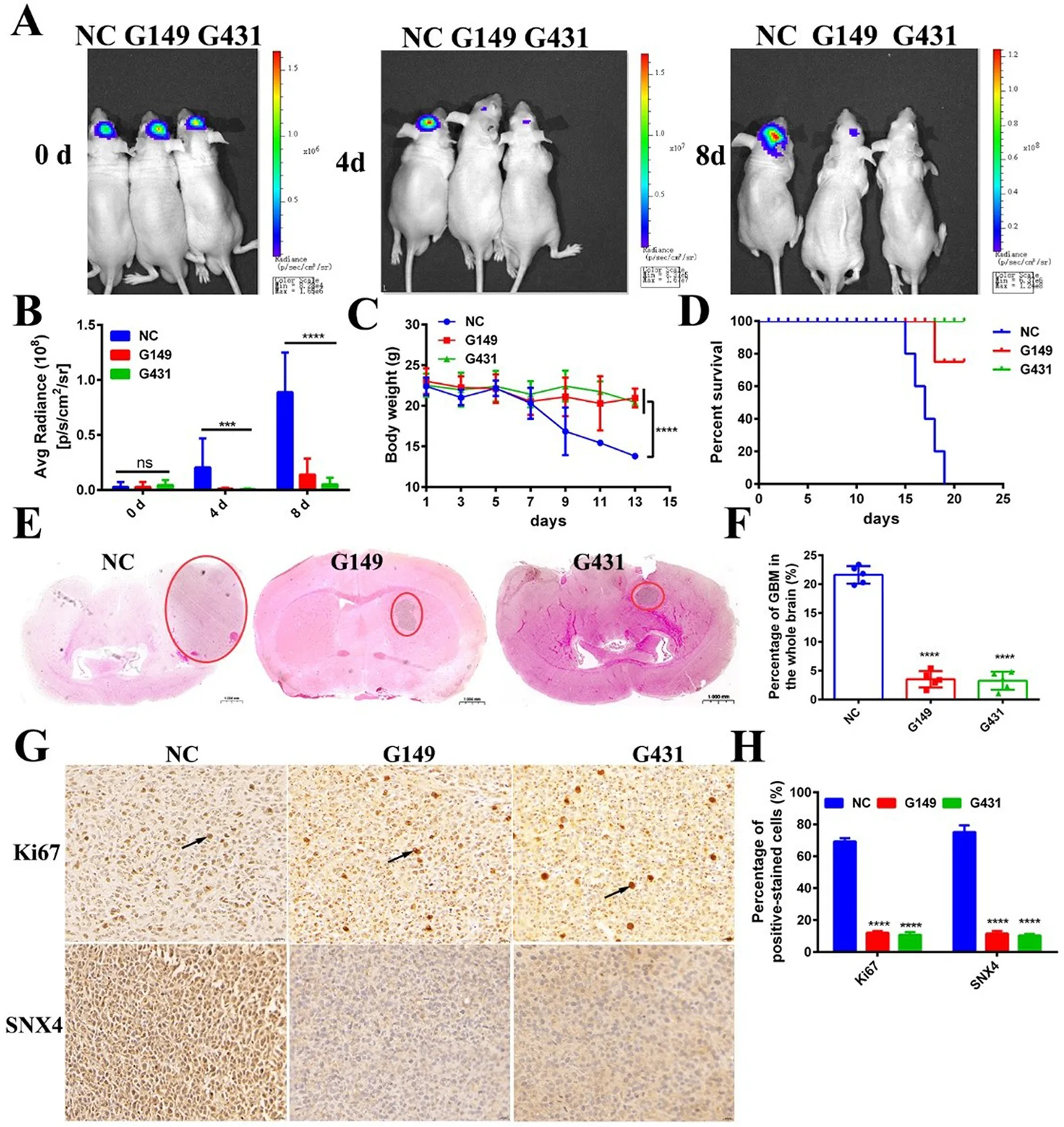
In vivo anti-GBM activity of candidate SNX4-binding compounds G149 and G431. (**A**) The representative bioluminescence images of the intracranial GBM tumor tissues in each indicated group mice taken on the day (day 0) before, on the fourth day after, and on the eighth day after treatment with G149 or G431 (*n* = 5 per group, only three mice shown). (**B**) Quantitative and statistical analyses of the bioluminescence intensity (shown as average radiance) from the luciferase-luciferin biochemical reaction occurred in the intracranial GBM tumor tissues in each group mice shown in the panel A. (**C**) The changes in body weight of mice measured every two days after treatment with G149 or G431 in each indicated group. (**D**) Survival curves of mice treated with G149 or G431 in each indicated group. (**E**) The representative photomicrographs of the HE staining results of the coronal sections of mouse brain tissues with the orthotopic GBM tissues (marked by a red oval) in each group. Scale bar, 2 mm. (**F**) The percentage of the area of the intracranial GBM tumor tissue in the whole area of the mouse brain in the panel E. (**G**) The representative photomicrographs of the IHC staining results showing the protein expression differences of SNX4 and Ki67 in the intracranial GBM tumor tissues in mouse brains in each group (*n* = 5 per group). (**H**) The quantitative and statistical analyses of the IHC staining results in the panel G. All data were expressed as the mean ± SEM of three independent experiments and statistically analyzed by Student’s t-test for P-values. Compared with NC, *P < 0.05; ** *P* < 0.01; *** *P* < 0.001; **** *P* < 0.0001. NC: negative control (drug vehicle control)

The in vivo anti-GBM effects of G149 and G431 were validated in the intracranial orthotopic GBM tumor mouse model. The results of live imaging showed that in the control group, the intracranial tumors gradually increased in size over time, with intracranial bioluminescence intensity reaching 10^8^ p/s/cm^2^/sr, while in the G149 and G431 treatment groups, the intracranial tumor bioluminescence did not increase after drug treatment, remained at 10^6^ p/s/cm^2^/sr (Fig. 6A-B, Fig S10A-C). Additionally, the body weight of the control group mice gradually decreased over time, whereas the body weight of the treatment group mice remained almost unchanged (Fig. 6C). All mice in the control group eventually died during this study, with a survival rate of 0%, while the G149 group mice had an 80% survival rate, and the G431 group mice had no deaths, with a survival rate of 100% (Fig. 6D). HE staining results indicated that the size of intracranial tumors in the control group mice was significantly larger, accounting for about 20% of the entire brain area, whereas in the treatment group mice, the size of intracranial tumors was much smaller, accounting for about 5% of the entire brain area (Fig. 6E-F, Fig S11). IHC staining results showed that the expression levels of Ki67 and SNX4 in the tumors of the control group mice were significantly higher than those in the treatment group (Fig. 6G-H, Fig S11). The results of HE staining of the paraffin sections from the indicated five major organs showed that G149 and G431 had almost no toxic effects on the five major organs of the mice (Fig S12). In summary, the two candidate SNX4-binding small-molecule compounds exhibit good anti-GBM tumor efficacies in mice.

### Candidate SNX4-binding compounds suppress TGFβ1/Smad signaling in GBM cells

**Fig. 7:**
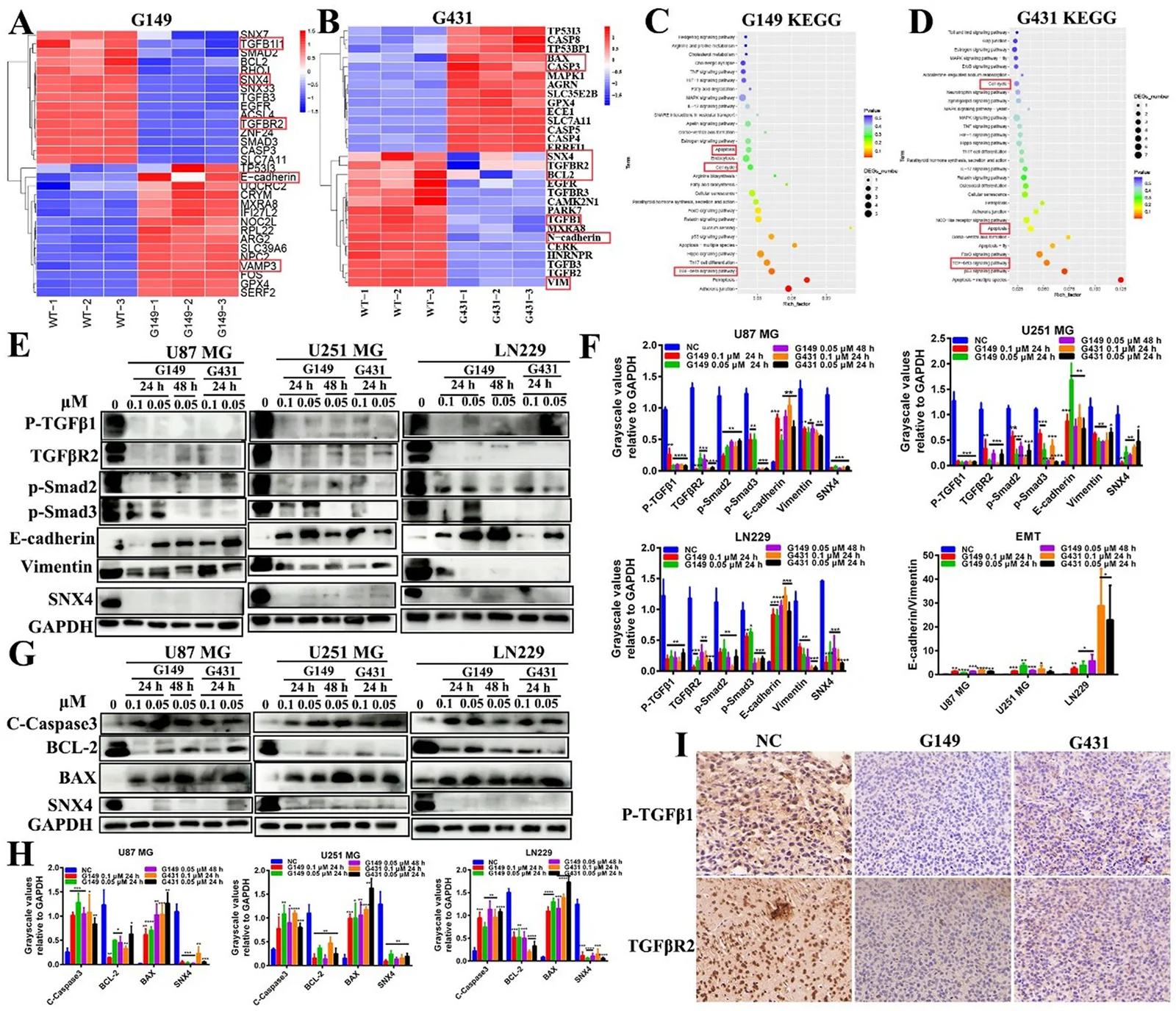
Exploration of the mechanism of action of two candidate SNX4-binding compounds G149 and G431 on GBM. (**A-B**) The differential gene enrichment analysis (shown as a heatmap by gene clustering) of the top 30 differentially expressed genes (DEGs) detected by RNA-Seq in U87 MG cells treated with G149 (**A**) and G431 (**B**), respectively. (**C-D**) The KEGG functional enrichment analysis showing the number of the DEGs detected by RNA-Seq enriched in the indicated biological processes in U87 MG cells treated with G149 (**C**) and G431 (**D**), respectively. (**E-F**) WB showing the effects of G149 and G431 on the expression levels and phosphorylation status of the four key component proteins in the TGFβ1/Smad pathway after GBM cells (U87 MG, U251 MG, and LN229) treated with the indicated concentrations of G149 or G431 (0.05 µM and 0.1 µM) for the indicated periods of time (24 h and 48 h) (**E**) and the quantitative and statistical analyses of the grayscale values of the protein bands in the panel E (**F**). (**G-H**) WB showing the effects of G149 and G431 on the expression levels of the three key component proteins in the apoptotic pathway after GBM cells (U87 MG, U251 MG and LN229) treated with the indicated concentrations of G149 or G431 (0.05 µM and 0.1 µM) for the indicated periods of time (24 h and 48 h) (**G**) and the quantitative and statistical analyses of the grayscale values of the protein bands in the panel G (**H**). (**I**) The representative photomicrographs of the IHC staining results of the protein expression differences of P-TGFβ1 and TGFβR2 in the intracranial GBM tumor tissues in mouse brain in each group. All data were expressed as the mean ± SEM of three independent experiments and statistically analyzed by Student’s t-test for P-values. Compared with NC, *P < 0.05; ** *P* < 0.01; *** *P* < 0.001; **** *P* < 0.0001. RNA-Seq: RNA sequencing or transcriptome sequencing

To explore the molecular mechanisms of small-molecule inhibitors targeting SNX4 on GBM, we performed transcriptome sequencing (RNA-Seq) on U87 MG cells treated with G149 or G431 for 24 h. Differential gene analysis revealed that G149 or G431 treatment significantly reduced SNX4 expression level, but increased the expression levels of BAX and CASP4 (Fig. 7A-B). Additionally, G149 or G431 treatment downregulated the expression of P-TGFβ1 and TGFβR2 (Fig. 7A-B). Among the EMT markers, G149 or G431 treatment upregulated E-cadherin gene and downregulated N-cadherin and Vimentin genes (Fig. 7A-B). These results are consistent with the RNA-Seq results of SNX4 knockdown cells described above in this study. KEGG enrichment analysis (Fig. 7C-D, Fig S13A, D) and GO enrichment analysis (Fig S13B-C, E-F) identified significant associations between SNX4 and the TGFβ1/Smad signaling pathway, cell cycle, and apoptosis. These findings are also consistent with the enrichment analysis results of SNX4 knockdown cells described above in this study. The combined analysis of the RNA-Seq data of both SNX4 knockdown cells and G149- and G431-treated cells revealed that more than 16,000 genes were the same in the three sets of sequencing data (Fig S13G), suggesting that G149 and G431 induce transcriptional changes that substantially overlap with those observed following SNX4 knockdown.

We further detected the expression levels of SNX4 and the major component proteins of the TGFβ1/Smad signaling pathway by WB in three GBM cell lines. We found that SNX4 was also hardly expressed in G149 and G431-treated GBM cells and P-TGFβ1, TGFβR2, p-Smad2, and p-Smad3 were significantly downregulated, or even barely detectable (Fig. 7E-F). These findings indicate that treatment with G149 or G431 is associated with reduced SNX4 expression and decreased activation of the TGFβ1/Smad signaling pathway. WB detection of EMT-related markers revealed an increased expression of E-cadherin and decreased expression of Vimentin in G149 and G431-treated GBM cell lines (Fig. 7E-F). The effect of two inhibitors on EMT-like process in intracranial GBM tissues of mice was further investigated by immunofluorescence staining. The results indicated an increased expression of E-cadherin and decreased expression of Vimentin in tumors from drug treatment mice, compared to control group mice (Fig S14A-B). These results suggest that G149 and G431 inhibit the EMT-like process in GBM in vivo. WB detection revealed that G149 or G431 treatment increased the expression of C-Caspase 3 and BAX, but decreased expression of BCL-2 in three GBM cell lines (Fig. 7G-H), suggesting that G149 and G431 promote apoptosis of GBM cells in vitro. The effect of two inhibitors on GBM apoptosis in vivo in intracranial GBM tissues of mice was studied by TUNEL staining. The results showed significantly increased cell apoptosis in tumors from inhibitor-treated mice, compared to control group mice (Fig S14C-D). This also further indicates that G149 and G431 promote apoptosis in GBM cells in vitro and in vivo. The effect of two inhibitors on the TGF-β1/Smad signaling pathway in intracranial GBM tissues of mice was further investigated by IHC staining. The results showed that G149 or G431 significantly downregulated expression of P-TGFβ1 and TGFβR2 in tumors from inhibitor-treated mice, compared to control group mice (Fig. 7I). In summary, candidate SNX4-binding small-molecule compounds exert their anti-GBM tumor role by inhibiting the TGFβ1/Smad signaling pathway.

## Discussion

Glioma cells have characteristics such as rapid proliferation, fast migration, strong invasiveness, and the ability to stimulate angiogenesis, leading to an invasive growth pattern of glioma and rapid progression to higher grades [15–17]. In clinical practice, due to the lack of specific molecular targets for GBM intervention and the presence of the BBB, it is difficult for molecularly targeted drugs and monoclonal antibodies to reach the GBM tissue [18, 19]. There is an urgent need to discover specific, novel molecules that regulate the occurrence, development, progression, and recurrence of GBM. These molecules may serve as key therapeutic targets [20–24]. To this end, by employing a set of approaches, especially virtual drug screening and molecular docking. In the present study, we demonstrated that SNX4 contributes to glioma progression and may serve as a potential prognostic biomarker for patients with glioma. In addition, we identified two candidate SNX4-binding small molecules that exhibited anti-glioma activity and recapitulated, at least in part, the phenotypic effects of SNX4 depletion. These findings provide a foundation for further mechanistic studies and support the future development and optimization of SNX4-targeting therapeutic strategies.

The SNXs are a large family involved in the regulation of protein transport and signal transduction [6, 7]. Dysregulated expressions of SNX family members are involved in various biological processes and disorders, including intracellular metabolic imbalance [6], impaired homeostatic responses [6], and the occurrence, development, metastasis, and drug resistance of tumors [6, 25]. A few members of SNX family have been studied in glioma. Pan-Cancer cohort analysis found that SNX29 was significantly upregulated in most tumor tissues including glioma and played an important role in the progression of various tumors, immune infiltration, and drug sensitivity [26]. SNX7 promotes glioblastoma cell proliferation, invasion, and survival by inhibiting protective autophagy [27]. SNX20 was highly expressed in glioma cell lines, and SNX20 knockdown significantly inhibited the proliferation and migration ability of glioma cells [28]. However, the mechanism of action of SNX family members such as SNX7, SNX20, and SNX29 in glioblastoma has not been elucidated. To our knowledge, the biological role of SNX4 in glioblastoma has not been previously investigated. In addition, Although the therapeutic potential of targeting SNX family proteins has recently attracted increasing attention, specific small-molecule modulators directly targeting SNX proteins remain largely unexplored.

SNX4 has been found to be associated with the progression of various tumors in recent years [29]. SNX4 is highly expressed in gastric cancer and negatively correlated with the survival of gastric cancer patients [10]. However, in clear cell renal cell carcinoma, the expression level of SNX4 is significantly lower than that in normal renal tissues, negatively correlated with histological grades, and positively correlated with the survival of patients [11]. Thus, the expression pattern of SNX4 in various types of tumors is not uniform. The mechanism of action of SNX4 and the regulatory mechanism of SNX4 expression in tumors are unclear, especially in gliomas. In this study, we demonstrated that SNX4 is highly expressed in GBM tissues and cell lines. Loss-of-function experiments showed that depletion of SNX4 significantly attenuated GBM cell proliferation, migration, invasion, EMT-like changes, and tumor growth while promoting apoptosis both in vitro and in vivo. Mechanistically, our data support a functional interaction between SNX4 and the TGFβ1/Smad signaling pathway. SNX4 depletion reduced the expression of TGFβ1 precursor, TGFβR2, p-Smad2, and p-Smad3, whereas pharmacological activation and inhibition of TGFβ signaling further indicated that this pathway contributes, at least in part, to the malignant phenotypes mediated by SNX4. Collectively, these findings identify SNX4 as a functionally important regulator of GBM progression and support its further investigation as a candidate therapeutic target for GBM.

One of the reasons for the limited outcome of GBM treatment is that the current drugs cannot cross the BBB and have severe side effects if a higher dosage is administered to achieve a certain degree of the BBB penetration [30]. Based on our findings above, we performed virtual screening to identify candidate small molecules predicted to bind SNX4 protein. Using various computer-assisted methods, in vitro cell-based drug activity screening, and in vivo nude mouse xenograft models, we identified G149 and G431 as two lead candidate compounds exhibiting potent anti-GBM activity among the screened top 50 candidate molecules. Our results also revealed that G149 and G431 were virtually non-toxic to the normal human astrocyte cell line, HA 1800, in vitro (Fig S6A-B, E-F; Fig S7A) and showed barely detectable side effects on the five major organs tested in vivo (Fig S12). Fluorescence imaging of NBD-labeled compounds showed a pattern of strong brain distribution following systemic administration. However, these observations should be interpreted cautiously because fluorescently labeled compounds may not fully reflect the pharmacokinetic behavior of the parental compounds. G149 is a CDK9 inhibitor (CDK9-IN-2) with anti-tumor effects through cell cycle regulation [31]. G431 (also called Prexasertib, LY2606368) is an inhibitor of checkpoint kinase 1 (CHK1) with potential anti-tumor activity. G431 (15 mg/kg) has anti-tumor effects on Calu-6 tumors xenografted in mice [32] and gastric cancer cells [33]. Mechanistically, G431 inhibits the proliferation of tumor cells by causing DNA damage and inhibiting the G2-M checkpoint in HeLa cells [32] and gastric cancer cells [33]. Therefore, the possibility that the anti-GBM activities of G149 and G431 are partially mediated through these previously reported targets cannot currently be excluded and warrants further investigation. Consistent with previous reports, treatment with G149 or G431 was associated with inhibition of the G2/M checkpoint in GBM cells. Whether these effects are mediated exclusively through SNX4 or also involve previously reported targets such as CDK9 or CHK1 remains to be determined. We found that the survival rate of mice in the G149-treated group was significantly lower than that of the G431-treated group. The stronger therapeutic efficacy observed for G431 may be related to its longer duration of detectable fluorescence signal in the brain compared with G149. However, because these observations were obtained using NBD-labeled compounds, they should be interpreted with caution, and future pharmacokinetic studies using unlabeled compounds and LC-MS/MS analysis will be required to validate their brain distribution and metabolic profiles. Although our computational and phenotypic data support SNX4 as a potential target of G149 and G431, additional target-engagement and pharmacodynamic studies will be required to establish their specificity and to exclude potential off-target mechanisms.

The TGFβ signaling pathway is activated in high grade gliomas, which promotes glioma cell growth and invasion and exacerbates the malignant behavior of gliomas [34, 35]. Recent studies have highlighted intracellular trafficking as a critical regulatory mechanism governing TGFβ signaling. Rather than being passively transported, TGFβ receptors continuously undergo endocytosis, recycling, and degradation, and alterations in these trafficking processes directly influence receptor abundance at the plasma membrane and the magnitude of downstream Smad activation. Dysregulation of receptor trafficking has therefore emerged as an important mechanism contributing to aberrant TGFβ signaling in cancer [36]. Consistent with this concept, SNX25 has been shown to negatively regulate TGFβ signaling by promoting receptor degradation [37], whereas a recent study demonstrated that ITGB5 promotes gastric cancer metastasis by recruiting SNX17 to facilitate endosomal recycling of TGFβR2, thereby preventing lysosomal degradation and sustaining TGFβ signaling [38]. In addition, SNX4 is a well-characterized endosomal sorting protein that regulates intracellular trafficking and recycling of several membrane-associated cargo proteins, including BACE1 and the transferrin receptor [39]. These findings collectively establish receptor trafficking as an important regulatory mechanism controlling TGFβ pathway activity. Given the established role of SNX4 in endosomal trafficking and membrane protein recycling, together with our observation that SNX4 depletion markedly reduced total TGFβR2 protein abundance, we speculate that SNX4 may similarly regulate TGFβ signaling by facilitating TGFβR2 recycling and/or preventing receptor degradation. Although this hypothesis requires direct experimental validation, it provides a biologically plausible explanation linking SNX4 to sustained TGFβ/Smad signaling in GBM. Meanwhile, the TGFβ/Smad signaling pathway is a classical signaling pathway that regulates EMT and significantly increases the invasiveness of GBM cells by promoting EMT-like processes, leading to chemotherapy tolerance and poor prognosis [40, 41]. However, it remains unknown how TGFβ1/Smad signaling pathway mediates the effects of novel genes involved in GBM and whether these novel genes in turn regulate TGFβ1/Smad signaling pathway. Our in vivo xenograft experiments demonstrated that treatment with G149 and G431 significantly inhibited intracranial GBM growth and was accompanied by reduced activation of the TGFβ1/Smad signaling pathway. The anti-GBM phenotypes induced by G149 and G431 were broadly similar to those observed following SNX4 knockdown, suggesting that these compounds may modulate common downstream signaling pathways. Furthermore, the results of the combined analysis of transcriptome sequencing revealed that more than 16,000 genes were identical in the three transcriptomes, indicating that these interventions share common transcriptional responses. These findings are consistent with the possibility that G149 and G431 modulate biological processes associated with SNX4; however, they do not by themselves establish SNX4 as the exclusive molecular target of these compounds, and contributions from previously reported targets cannot be excluded. Mechanistic analyses showed that both SNX4 depletion and treatment with G149 or G431 were associated with reduced activation of the TGFβ1/Smad signaling pathway. Our genetic and pharmacological studies support a functional interaction between SNX4 and the TGFβ1/Smad signaling pathway during GBM progression. G149 and G431 suppressed TGFβ/Smad signaling and attenuated malignant phenotypes in GBM cells. Although these findings are consistent with modulation of SNX4-associated signaling, additional studies will be required to determine the extent to which these effects are directly mediated through SNX4. Furthermore, our newly performed pharmacological inhibition experiments demonstrated that Galunisertib markedly attenuated SNX4 overexpression-induced Smad activation and malignant phenotypes, further supporting a functional interaction between SNX4 and the TGFβ1/Smad signaling pathway. Collectively, our genetic and pharmacological evidence indicates that activation of the TGFβ1/Smad pathway contributes, at least in part, to SNX4-mediated GBM progression, given the established role of SNX4 in endosomal trafficking and membrane protein recycling, together with previous reports demonstrating that sorting nexin-dependent trafficking regulates TGFβ receptor turnover and signaling, we speculate that SNX4 may facilitate the recycling of internalized TGFβR2 back to the plasma membrane and/or prevent its lysosomal degradation, thereby maintaining receptor abundance and sustaining TGFβ/Smad signaling. Future studies employing receptor trafficking assays, including surface biotinylation, receptor internalization and recycling analyses, and lysosomal colocalization studies, will be required to determine whether TGFβR2 is a direct cargo of SNX4 and to elucidate the underlying molecular mechanism.

Our current study has some limitations. First, although G149 and G431 have previously been reported as inhibitors of CDK9 and CHK1, respectively, we did not directly evaluate the contribution of these previously reported targets to their anti-GBM activities. Therefore, we cannot exclude the possibility that the observed biological effects are partially mediated through additional molecular targets besides SNX4. Second, although our data suggest that G149 and G431 reduce SNX4 protein expression and suppress SNX4-associated signaling, the molecular mechanisms underlying these effects remain unclear. Interestingly, our quantitative RT-PCR analysis demonstrated that G149 and G431 did not significantly affect SNX4 mRNA expression, whereas G149 and G431 markedly reduced SNX4 protein abundance. These findings suggest that these compounds regulate SNX4 primarily through a post-transcriptional mechanism rather than transcriptional repression. However, the precise post-transcriptional mechanism by which G149 and G431 reduce SNX4 protein abundance has not yet been elucidated. Whether this effect also results from altered protein stability and/or degradation, such as ubiquitin-proteasome-mediated degradation, lysosomal degradation, or other post-translational regulatory mechanisms remains to be determined. Future studies employing cycloheximide chase assays, proteasome and lysosome inhibition experiments, and ubiquitination analyses will be required to determine whether these compounds regulate SNX4 protein stability and/or degradation. Third, brain distribution was evaluated using NBD-labeled derivatives of G149 and G431 rather than the unlabeled parental compounds. Although fluorescence imaging suggests brain accumulation after systemic administration, these results should be interpreted with caution because conjugation with NBD may alter the physicochemical properties of the compounds, and the fluorescent signal cannot distinguish intact compounds from their metabolites or nonspecific tissue accumulation. Future pharmacokinetic studies using unlabeled compounds and LC-MS/MS-based tissue distribution analyses will be necessary to validate BBB penetration. Fourth, it is known that glioma can disrupt the integrity of the BBB at its location. We examined the BBB-crossing ability of G149-NBD or G431-NBD in nude mice with or without bearing intracranial orthotopic GBM tumor. Our results are similar in these two GBM mice models (Fig S8D-E vs. Fig S8G-H; Fig S9D-E vs. Fig S9G-H), suggesting that G149-NBD and G431-NBD themselves possess the good intrinsic BBB-crossing ability, irrespective of the integrity of the BBB. Fifth, the adjacent brain tissues used as controls in this study may not fully represent normal brain tissue because peritumoral regions can already exhibit molecular alterations associated with the tumor microenvironment. Future studies incorporating normal brain tissues from non-neoplastic donors will further strengthen our observations. Finally, although our genetic and pharmacological studies support a functional interaction between SNX4 and the TGFβ1/Smad signaling pathway, the precise molecular mechanism linking SNX4 to TGFβ receptor trafficking or signaling remains to be elucidated. Future studies investigating receptor recycling, membrane localization, and endosomal trafficking will further clarify this mechanism.

To our knowledge, this study demonstrates that SNX4 is highly expressed in GBM and may serve as a potential prognostic biomarker. We identified two candidate SNX4-binding small molecules with anti-GBM activity and showed that genetic manipulation of SNX4 level together with pharmacological activation and inhibition of the TGFβ pathway consistently supports a functional interaction between SNX4 and TGFβ1/Smad signaling during GBM progression. Collectively, these findings establish a framework for further mechanistic studies and support the future development and optimization of SNX4-directed therapeutic strategies for GBM.

## Supplementary Information

Below is the link to the electronic supplementary material.

Supplementary Material 1

## Data Availability

All original data and materials will be made available upon a reasonable request.

## References

[1] Schaff LR, Mellinghoff IK. Glioblastoma and Other Primary Brain Malignancies in Adults: A Review. JAMA. 2023;329(7):574–87. 10.1001/jama.2023.0023 PMC11445779 36809318

[2] Tsien CI, Pugh SL, Dicker AP, Raizer JJ, Matuszak MM, Lallana EC, et al. NRG Oncology/RTOG1205: A Randomized Phase II Trial of Concurrent Bevacizumab and Reirradiation Versus Bevacizumab Alone as Treatment for Recurrent Glioblastoma. J Clin Oncol. 2023;41(6):1285–95. 10.1200/JCO.22.00164 PMC9940937 36260832

[3] Karschnia P, Gerritsen JKW, Teske N, Cahill DP, Jakola AS, van den Bent M, et al. The oncological role of resection in newly diagnosed diffuse adult-type glioma defined by the WHO 2021 classification: a Review by the RANO resect group. Lancet Oncol. 2024;25(9):e404–19. 10.1016/S1470-2045(24)00130-X 39214112

[4] Zhang X, Tan Y, Li T, Tan D, Fu B, Yang M, et al. Intercellular adhesion molecule-1 suppresses TMZ chemosensitivity in acquired TMZ-resistant gliomas by increasing assembly of ABCB1 on the membrane. Drug Resist Updat. 2024;76:101112. 10.1016/j.drup.2024.101112 38924997

[5] Wu W, Klockow JL, Zhang M, Lafortune F, Chang E, Jin L, et al. Glioblastoma multiforme (GBM): An overview of current therapies and mechanisms of resistance. Pharmacol Res. 2021;171:105780. 10.1016/j.phrs.2021.105780 PMC8384724 34302977

[6] Vieira N, Rito T, Correia-Neves M, Sousa N. Sorting Out Sorting Nexins Functions in the Nervous System in Health and Disease. Mol Neurobiol. 2021;58(8):4070–106. 10.1007/s12035-021-02388-9 PMC8280035 33931804

[7] Cullen PJ, Korswagen HC. Sorting nexins provide diversity for retromer-dependent trafficking events. Nat Cell Biol. 2011;14(1):29–37. 10.1038/ncb2374 PMC3613977 22193161

[8] Worby CA, Dixon JE. Sorting out the cellular functions of sorting nexins. Nat Rev Mol Cell Biol. 2002;3(12):919–31. 10.1038/nrm974 12461558

[9] Poppinga J, Barrett NJ, Cornelisse LN, Verhage M, van Weering JRT. Endosomal sorting protein SNX4 limits synaptic vesicle docking and release. Elife. 2024;13:RP97910. 10.7554/eLife.97910 PMC11658762 39699951

[10] Hu B, Yin G, Sun X. Identification of specific role of SNX family in gastric cancer prognosis evaluation. Sci Rep. 2022;12(1):10231. 10.1038/s41598-022-14266-y PMC9205943 35715463

[11] Chai YM, Zhou ZB, Liu RZ, Cui YS, Zhang Y. SNX4 Is Correlated With Immune Infiltration and Prognosis in Clear Cell Renal Cell Carcinoma. World J Oncol. 2024;15(5):809–24. 10.14740/wjon1868 PMC11424112 39328330

[12] Yamada K, Motohashi S, Oikawa T, Tago N, Koizumi R, Ono M, et al. Extended-synaptotagmin 1 engages in unconventional protein secretion mediated via SEC22B(+) vesicle pathway in liver cancer. Proc Natl Acad Sci U S A. 2022;119(36):e2202730119. 10.1073/pnas.2202730119 PMC9457520 36044553

[13] Yao Z, Zhang F, Qi C, Wang C, Mao M, Zhao C, et al. SECTM1 promotes the development of glioblastoma and mesenchymal transition by regulating the TGFbeta1/Smad signaling pathway. Int J Biol Sci. 2024;20(1):78–93. 10.7150/ijbs.84591 PMC10750278 38164182

[14] Sastry GM, Adzhigirey M, Day T, Annabhimoju R, Sherman W. Protein and ligand preparation: parameters, protocols, and influence on virtual screening enrichments. J Comput Aided Mol Des. 2013;27(3):221–34. 10.1007/s10822-013-9644-8 23579614

[15] Kampers LFC, Metselaar DS, Vinci M, Scirocchi F, van Veldhuijzen S, Eyrich M et al. The Complexity of Malignant Glioma Treatment. Cancers (Basel). 2025;17(5):879. 10.3390/cancers17050879 PMC11899524 40075726

[16] Wu X, Fu M, Ge C, Zhou H, Huang H, Zhong M, et al. m(6)A-Mediated Upregulation of lncRNA CHASERR Promotes the Progression of Glioma by Modulating the miR-6893-3p/TRIM14 Axis. Mol Neurobiol. 2024;61(8):5418–40. 10.1007/s12035-023-03911-w 38193984

[17] Wen Y, Zhou W, Zhao Z, Ma D, Mao J, Cai Y, et al. Annexin A’s Life in Pan-Cancer: Especially in Glioma Immune Cells. Neuromolecular Med. 2025;27(1):17. 10.1007/s12017-024-08827-9 40011350

[18] Pouyan A, Ghorbanlo M, Eslami M, Jahanshahi M, Ziaei E, Salami A, et al. Glioblastoma multiforme: insights into pathogenesis, key signaling pathways, and therapeutic strategies. Mol Cancer. 2025;24(1):58. 10.1186/s12943-025-02267-0 PMC11863469 40011944

[19] Lan Z, Li X, Zhang X, Glioblastoma. An Update in Pathology, Molecular Mechanisms and Biomarkers. Int J Mol Sci. 2024;25(5):3040 . 10.3390/ijms25053040 PMC10931698 38474286

[20] Muir M, Gopakumar S, Traylor J, Lee S, Rao G. Glioblastoma multiforme: novel therapeutic targets. Expert Opin Ther Targets. 2020;24(7):605–14. 10.1080/14728222.2020.1762568 32394767

[21] Aldape K, Zadeh G, Mansouri S, Reifenberger G, von Deimling A. Glioblastoma: pathology, molecular mechanisms and markers. Acta Neuropathol. 2015;129(6):829–48. 10.1007/s00401-015-1432-1 25943888

[22] Qi M, Sun LA, Zheng LR, Zhang J, Han YL, Wu F, et al. Expression and potential role of FOSB in glioma. Front Mol Neurosci. 2022;15:972615. 10.3389/fnmol.2022.972615 PMC9597691 36311014

[23] Yang H, Zhou H, Fu M, Xu H, Huang H, Zhong M, et al. TMEM64 aggravates the malignant phenotype of glioma by activating the Wnt/beta-catenin signaling pathway. Int J Biol Macromol. 2024;260(Pt 1):129332. 10.1016/j.ijbiomac.2024.129332 38232867

[24] Zhu X, Wu X, Yang H, Xu Q, Zhang M, Liu X, et al. m(6)A-mediated upregulation of LINC01003 regulates cell migration by targeting the CAV1/FAK signaling pathway in glioma. Biol Direct. 2023;18(1):27. 10.1186/s13062-023-00386-6 PMC10239147 37270527

[25] Yang L, Tan W, Yang X, You Y, Wang J, Wen G et al. Sorting nexins: A novel promising therapy target for cancerous/neoplastic diseases. J Cell Physiol. 2021; 236: 3317–35. 10.1002/jcp.30093 33090492

[26] Xu C, Li F, Liu Z, Yan C, Xiao J. Pan-cancer analysis of the prognostic and immunological role of SNX29: a potential target for survival and immunotherapy. BMC Med Genomics. 2023;16(1):34. 10.1186/s12920-023-01466-2 PMC9951530 36829159

[27] Zhang Y, Chen B, Liu R, Mei W, Lin Y. Deciphering glioblastoma pathogenesis: Insights from mitophagy dysregulation and SNX7 as a therapeutic target. Brain Res Bull. 2025;220:111184. 10.1016/j.brainresbull.2024.111184 39736337

[28] Chen X, Jiang X, Wang H, Wang C, Wang C, Pan C, et al. DNA methylation-regulated SNX20 overexpression correlates with poor prognosis, immune cell infiltration, and low-grade glioma progression. Aging. 2022;14(12):5211–22. 10.18632/aging.204144 PMC9271302 35771139

[29] Skanland SS, Walchli S, Brech A, Sandvig K. SNX4 in complex with clathrin and dynein: implications for endosome movement. PLoS ONE. 2009;4(6):e5935. 10.1371/journal.pone.0005935 PMC2691479 19529763

[30] Sadowski K, Jazdzewska A, Kozlowski J, Zacny A, Lorenc T, Olejarz W. Revolutionizing Glioblastoma Treatment: A Comprehensive Overview of Modern Therapeutic Approaches. Int J Mol Sci. 2024;25(11):5774. 10.3390/ijms25115774 PMC11172387 38891962

[31] Panagiotou E, Gomatou G, Trontzas IP, Syrigos N, Kotteas E. Cyclin-dependent kinase (CDK) inhibitors in solid tumors: a review of clinical trials. Clin Transl Oncol. 2022;24(2):161–92. 10.1007/s12094-021-02688-5 34363593

[32] King C, Diaz HB, McNeely S, Barnard D, Dempsey J, Blosser W, et al. LY2606368 Causes Replication Catastrophe and Antitumor Effects through CHK1-Dependent Mechanisms. Mol Cancer Ther. 2015;14(9):2004–13. 10.1158/1535-7163.MCT-14-1037 26141948

[33] Yin Y, Shen Q, Zhang P, Tao R, Chang W, Li R, et al. Chk1 inhibition potentiates the therapeutic efficacy of PARP inhibitor BMN673 in gastric cancer. Am J Cancer Res. 2017;7(3):473–83. PMC5385637 28401005

[34] Mao J, Sun Z, Cui Y, Du N, Guo H, Wei J, et al. PCBP2 promotes the development of glioma by regulating FHL3/TGF-beta/Smad signaling pathway. J Cell Physiol. 2020;235(4):3280–91. 10.1002/jcp.29104 PMC7166520 31693182

[35] Chen Y, Di C, Zhang X, Wang J, Wang F, Yan JF, et al. Transforming growth factor beta signaling pathway: A promising therapeutic target for cancer. J Cell Physiol. 2020;235(3):1903–14. 10.1002/jcp.29108 31332789

[36] Yakymovych I, Yakymovych M, Heldin CH. Intracellular trafficking of transforming growth factor beta receptors. Acta Biochim Biophys Sin (Shanghai). 2018;50(1):3–11. 10.1093/abbs/gmx119 29186283

[37] Hao X, Wang Y, Ren F, Zhu S, Ren Y, Jia B, et al. SNX25 regulates TGF-beta signaling by enhancing the receptor degradation. Cell Signal. 2011;23(5):935–46. 10.1016/j.cellsig.2011.01.022 21266196

[38] Lei T, Lin Y, Lai X, Zhang Y, Ma Y, Wang X, et al. ITGB5 facilitates gastric cancer metastasis by promoting TGFBR2 endosomal recycling. Cancer Lett. 2024;592:216953. 10.1016/j.canlet.2024.216953 38729557

[39] Kim NY, Cho MH, Won SH, Kang HJ, Yoon SY, Kim DH. Sorting nexin-4 regulates beta-amyloid production by modulating beta-site-activating cleavage enzyme-1. Alzheimers Res Ther. 2017;9(1):4. 10.1186/s13195-016-0232-8 PMC5251330 28109317

[40] Zhang Z, Rohweder PJ, Ongpipattanakul C, Basu K, Bohn MF, Dugan EJ, et al. A covalent inhibitor of K-Ras(G12C) induces MHC class I presentation of haptenated peptide neoepitopes targetable by immunotherapy. Cancer Cell. 2022;40(9):1060–9. e7. 10.1016/j.ccell.2022.07.005 PMC10393267 36099883

[41] He L, Yang H, Zhu XL, Zhang Y, Lv K. Knockdown of long non-coding RNA SLC8A1-AS1 attenuates cell invasion and migration in glioma via suppression of Wnt/beta-catenin signaling pathways. Brain Res Bull. 2021;176:112–20. 10.1016/j.brainresbull.2021.08.011 34474120

