# Supplementary material for: SNX4 promotes glioblastoma progression through the TGFβ1/Smad signaling pathway and represents a potential therapeutic target
Source: J Transl Med. 2026 Aug 11;24:1408. doi: 10.1186/s12967-026-08784-w (PMC13746591; doi:10.1186/s12967-026-08784-w)
Supplement: Supplementary file 1 [file 12967_2026_8784_MOESM1_ESM.docx]

**SNX4 promotes glioblastoma progression through the TGFβ1/Smad signaling pathway and represents a potential therapeutic target**

Zhipeng Yao^1,2^, Pengcheng Xu^1,2^, Tianxiao Shen^1^, Yunpeng Hua^1^, Shaodong Ding^1^, Yan Xu^1^, Wenhua Zhong^1,2^, Xinya Zhao^2^, Shan Jiang^4^, Min Qi^1,2^, Xiaochun Jiang^1,2,3^*, Shizhang Ling^1,2,3^*

^1^ The Translational Research Institute for Neurological Disorders, Department of Neurosurgery, the First Affiliated Hospital of Wannan Medical University (Yijishan Hospital of Wannan Medical University), Wuhu, Anhui, China.

^2^ The Institutes of Brain Science, Wannan Medical University, Wuhu, Anhui, China.

^3^ Anhui Province Digital Brain Engineering Research Center, Wannan Medical University, Wuhu, Anhui, China.

^4^ The School of Basic Medical Sciences, Wannan Medical University, Wuhu, Anhui, China.

^*^ Corresponding authors: [sling@wnmc.edu.cn](mailto:sling@wnmc.edu.cn) or [lingsz@hotmail.com](mailto:lingsz@hotmail.com) (S.L.); [jiangxiaochun2001@hotmail.com](mailto:jiangxiaochun2001@hotmail.com) (X.J.)

# **Supporting information**

# **1**. **Supplementary Materials and Methods**

# **2. Supplementary Results**

# **1. Supplementary Materials and Methods**

# **Reagents and Antibodies**

Dulbecco's Modified Eagle Medium (DMEM) were purchased from Key Biotech (KGM12800-500, Nanjing, China). Fetal bovine serum (FBS, F8318-500 ml, Sigma-Aldrich, Shanghai, China), Phosphate Buffered Saline (PBS) was purchased from Servicebio (G4250-500 ml, Wuhan, China), Trypsin was purchased from Gibco (0.25% Trypsin-EDTA (1X), 25200-072). Puromycin (T2219, TargetMol, USA). TRIzol (10296-010, Invitrogen, Carlsbad, CA, USA). RNA extraction kit (DP431, Tiangen Biotech, Beijing, China). cDNA synthesis kit (KR116, Tiangen Biotech, Beijing, China). SuperReal PreMix Plus SYBR Green kit (FP205, Tiangen Biotech, Beijing, China). BCA kit (P0010, Beyotime Technology, Shanghai, China). SNX4 polyclonal antibody (1:1000, Affinity Biosciences Cat# DF4538, RRID: AB_2836889, USA), TGFβ1 polyclonal antibody ( that recognizes TGFβ1 precusor,1:2000, Proteintech Cat# 21898-1-AP, 44 kDa, RRID: AB_2811115, Wuhan, China), TGFβR2 monoclonal antibody (1:5000 dilution, Proteintech Cat# 66636-1-Ig, RRID: AB_2881995, Wuhan, China), p-Smad2 (phosphor-S467) antibody (1:1000, Abcam Cat# ab280888, RRID: AB_3099532, Cambridge, UK), p-Smad3 (phosphor-S423+S425) antibody (1:2000, Abcam Cat# ab52903, RRID: AB_882596, Cambridge, UK), E-cadherin polyclonal antibody (1:10000, Proteintech Cat# 20874-1-AP, RRID: AB_10697811, Wuhan, China), Vimentin monoclonal antibody (1:50000, Proteintech Cat# 60330-1-Ig, RRID: AB_2881439, Wuhan, China), cleaved Caspase-3 (1:500, Abcam Cat# ab32042, RRID: AB_725947), BCL-2 (1:2000, Abcam Cat# ab182858, RRID: AB_2715467), BAX (1:5000, Abcam Cat# ab32503, RRID: AB_725631, Cambridge, UK), GAPDH Monoclonal antibody (1:50000, Proteintech Cat# 60004-1-Ig, RRID: AB_2107436, Wuhan, China). HRP-labeled goat anti-mouse IgG (H+ L) secondary antibody (1:5000, Proteintech Cat# SA00001-1, RRID: AB_2722565, Wuhan, China), HRP-labeled goat anti-rabbit IgG secondary antibody (1:5000, Abcam Cat# ab6721, RRID: AB_955447, Cambridge, UK). ECL detection kit (KeyGen Biotech, Nanjing, China). Goat anti-rabbit IgG H&L (Alexa Fluor® 594) (1:500, Abcam Cat# ab150080, RRID: AB_2650602, Cambridge, UK), Goat anti-mouse IgG H&L (Alexa Fluor® 488) (1:500, Abcam Cat# ab150113, RRID: AB_2576208, Cambridge, UK). DAPI (P0131 Beyotime Technology, Shanghai, China). IHC staining kit (Kgos60, KeyGen Biotech, Nanjing, China). Cell Counting Kit-8 (CCK-8, C0005, TargetMol, USA). kFluor488-EdU cell proliferation assay kit (KGA331, KeyGen Biotech, Nanjing, China). Cell cycle detection kit (KGA512, KeyGen Biotech, Nanjing, China). Matrigel (1:3, KGL5104-5, KeyGen Biotech, Nanjing, China). Apoptosis Detection Kit (KGA1107-50, KeyGen Biotech, Nanjing, China). G149 (T14918, TargetMol, USA), G431 (T4310, TargetMol, USA), 4-Chloro-7-nitrobenzoxadiazole (NBD-Cl, NBD chloride, T19030, TargetMol, USA). Galunisertib (LY2157299, T2510, TargetMol, USA). TUNEL kit (12,156,792,910, Roche, Switzerland). DAPI (P0131, Beyotime Technology, Shanghai, China).

# **Real-time fluorescent quantitative reverse transcription PCR (RT-qPCR)**

In this study, RT-qPCR was used to detect the expression level of SNX4 in GBM cell lines and clinical GBM tissue samples, in stable SNX4 knockdown GBM cell lines for the knockdown efficiency of the SNX4, and the expression levels of TGFβ1 precursor (P-TGFβ1) and TGFβR2 in GBM xenograft tissues from nude mice experiments. When the cell density reached approximately 80-90%, total RNA was extracted from HA 1800 and glioma cell lines (U87 MG, U251 MG, LN229, U87 MG-shNC, U87 MG-sh1, U87 MG-sh2, U251 MG-shNC, U251 MG-sh1, U251 MG-sh2, LN229-shNC, LN229-sh1, LN229-sh2) using the TRIzol method (10296-010, Invitrogen, Carlsbad, CA, USA). Total RNA extraction from tissues followed the protocol of the RNA extraction kit. The total RNA was reverse-transcribed to cDNA using the cDNA synthesis kit, and finally, the RT-qPCR detection was performed using the SuperReal PreMix Plus SYBR Green kit. The primer sequences are as follows:

GAPDH forward: 5′-CAACTACATGGTTTACATGTTC-3′,

GAPDH reverse: 5′-GCCAGTGGACTCCACGAC-3′;

SXN4 forward: 5′-TGGAAGAAATGCCATGAACA-3′,

SNX4 reverse: 5′-GCAGAGGTGGCACAACAATA-3′;

TGFβ1 forward: 5′-GGCCTTTCCTGCTTCTCAT-3′,

TGFβ1 reverse: 5′-GTCCTTGCGGAAGTCAATGT-3′.

TGFβR2 forward: 5′-TGTGAGATTTTCCACCTGTGACA-3′,

TGFβR2 reverse: 5′-ATGCACTTTGGAGAAGCAGC-3′.

# **Western blotting (WB)**

The WB was used to detect expression level of SNX4 in the GBM cell lines and clinical GBM tissue samples, the knockdown efficiency of SNX4 in GBM cell lines, apoptosis pathway-related proteins, and the expression of related proteins in signaling pathways. Total protein was extracted from HA1800 and glioma cell lines (U87 MG, U251 MG, LN229, U87 MG-shNC, U87 MG-sh1, U87 MG-sh2, U251 MG-shNC, U251 MG-sh1, U251 MG-sh2, LN229-shNC, N229-sh1, LN229-sh2,) and clinical GBM tissue samples using RIPA lysis buffer, followed by protein quantification using the BCA kit. After separation by 10% SDS-PAGE gel, the resolved proteins were transferred to polyvinylidene fluoride (PVDF) membrane. The PVDF membrane was blocked with 5% skim milk at room temperature for 2 h, then incubated with each of the indicated primary antibodies overnight at the indicated dilution. These primary antibodies are SNX4 polyclonal antibody, TGFβ1 polyclonal antibody, TGFβR2 monoclonal antibody, p-Smad2 antibody, p-Smad3 antibody, E-cadherin polyclonal antibody, Vimentin monoclonal antibody, cleaved Caspase-3, BCL-2, BAX, and GAPDH Monoclonal antibody. The PVDF membrane was washed 3-5 times with TBST for 10 min each time and then incubated with HRP-conjugated goat anti-mouse IgG (H+ L) secondary antibody or HRP-conjugated goat anti-rabbit IgG secondary antibody for 1 h at room temperature. The PVDF membrane was washed 3-5 times with TBST for 10 min each time. Finally, the chemiluminescent signal of the target protein was detected using ECL detection kit, and visualized using ImageQuant 800 (GE, USA). The grayscale value of the band was analyzed using ImageJ software.

# **Immunofluorescence staining (IF)**

The IF was used to the expression of SNX4 in GBM cell lines and clinical GBM tissue samples, as well as EMT markers in GBM tissues from animal treatment experiments. Cells (HA 1800, U87 MG, U251 MG, LN229) were seeded at a density of 1×10^5^ cells per well into a 96-well culture plate and cultured in a cell incubator for 24 h. They were then fixed with 4% formaldehyde for 15 min, washed three times with PBS, and blocked with 5% BSA for 2 h. Incubated with SNX4 antibody overnight at 4°C. After washing three times with PBS, incubated with secondary antibody goat anti-rabbit IgG H&L (Alexa Fluor® 594) for 1 h at room temperature, washed three times with PBS. Finally, stained with DAPI for 10 min, and photographed a fluorescence microscope (Carl Zeiss Axio Scope Al, Germany). Clinical tissue paraffin sections and animal GBM tissue paraffin sections were dewaxed in xylene and rehydrated in graded alcohols, blocked with 5% BSA for 2 h, and incubated overnight at 4°C with SNX4 antibody, E-cadherin polyclonal antibody, or Vimentin monoclonal antibody. After three washes with PBS, sections were incubated with goat anti-rabbit IgG H&L (Alexa Fluor® 594) or goat anti-mouse IgG H&L (Alexa Fluor® 488) at room temperature for 1 hour, washed three times with PBS. Finally, tissue sections were coverslipped with anti-fluorescence quenching sealing solution containing DAPI, scanned by 3D HISTECH digital pathology system (Pannoramic MIDI II, RRID: SCR_024834, Hungary), and analyzed with ImageJ software.

# **Immunohistochemical staining (IHC)**

The IHC was used to detect expression levels of SNX4 in clinical GBM tissue samples and the related proteins in the signaling pathways in the xenograft GBM tissues in nude mouse brains. Paraffin tissue sections were dewaxed in xylene and rehydrated in a series of graded alcohols. The tissue sections were submerged in 0.1 M citrate buffer (pH 6.0) and boiled for 15 min for antigen retrieval, then incubated with 3% H_2_O_2_ for 10 min in the darkroom to quench the endogenous peroxidase activity, and followed by incubation with PBS with 5% BSA for 30 min at room temperature to block non-specific binding sites in tissue sections. Each set of the tissue sections was then incubated with each of the indicated primary antibodies, which are SNX4 antibody, TGFβ1 polyclonal antibody, TGFβR2 monoclonal antibody, or Ki67 polyclonal antibody overnight at 4°C, then washed with PBS for 10 min each time for three times, and incubated with the HRP-conjugated secondary antibody provided in the IHC staining kit for 1 h at room temperature. After washing with PBS for 10 min each time for three times, the tissue sections were incubated with DAB solution provided in the IHC staining kit for up to 10 min. After washed with tap water, the tissue sections were counterstained with hematoxylin solution, dehydrated in graded alcohols, and cleared in xylene, followed by coverslipping with anti-fading sealant solution. The stained tissue sections were scanned by 3D HISTECH digital pathology system (Pannoramic MIDI II, RRID: SCR_024834, Hungary) and analyzed with ImageJ software.

# **CCK8 assay**

The effects of SNX4 knockdown on cell proliferation and the effects of small molecule inhibitors targeting SNX4 on cell activity were examined in U87 MG, U251 MG, and LN229 cell lines by Cell Counting Kit-8. Stable SNX4 knockdown U87 MG, U251 MG or LN229 cell lines and their control cell lines were inoculated in 96-well cell plates at a density of 2×10^3^ cells per well. After 24 h of cell culture, 10 µL of CCK-8 solution was added to each well with an incubation for 1 h, then the absorbance at 450 nm was measured using a microplate reader (Epoch 2, BioTek, RRID: SCR_019740, USA). Subsequently, cell proliferation was assayed every 24 h for five consecutive days and cell proliferation curves were plotted. U87 MG, U251 MG or LN229 cell lines were inoculated in 96-well cell plates at a density of 1×10^4^ cells per well, and the cells were cultured for 24 h. Targeted SNX4 small molecule inhibitors, G149 and G431, were diluted at each of the two different concentrations and added to the cells, and the activity of the cells was assayed by CCK-8 at 24 h and 48 h intervals.

# **5-Ethynyl-2'-deoxyuridine (EdU) assay**

The EdU solution was used to detect the effect of SNX4 knockdown on cell proliferation and the effect of small molecule inhibitors targeting SNX4 on cell proliferation in U87 MG, U251 MG, and LN229 cell lines. Stable SNX4 knockdown U87 MG, U251 MG or LN229 cell lines and their control cell lines were inoculated at a density of 1×10^4^ cells per well in 96-well cell plates and cultured for 24 h. U87 MG, U251 MG or LN229 cell lines were inoculated at a density of 1×10^4^ cells per well in 96-well cell plates and cultured for 24 h. Each of the targeted SNX4 small molecule inhibitors, G149 and G431, was added to the cells at each of the two different concentrations and incubated for 24 h. Cells were stained with a kFluor488-EdU cell proliferation assay kit at a working concentration of 50 µM of EdU and then incubated for 2 h after addition of EdU. The cells were observed under a fluorescence microscope (Axio Scope Al, Carl Zeiss, Germany) and photographed. The percentage of cell proliferation was measured using ImageJ software (RRID: SCR_003070).

# **Cell cycle assay**

The effects of SNX4 knockdown on the cell cycle and the effects of small molecule inhibitors targeting SNX4 on the cell cycle were examined in U87 MG, U251 MG, and LN229 cell lines by flow cytometry. Stable SNX4 knockdown U87 MG, U251 MG or LN229 cell lines and their control cell lines were seeded in 6-well cell plates at a density of 5×10^5^ cells per well and cultured for 24 h. U87 MG, U251 MG or LN229 cell lines were inoculated at a density of 5×10^5^ cells per well in 6-well cell plates and were added with each of the SNX4 small molecule inhibitors, G149 and G431, at each of the two concentrations, and incubated for 24 h and 48 h. Cells were collected in 1.5 ml of 70% pre-cooled ethanol and fixed at 4°C overnight. Cells were processed with a cell cycle detection kit. Analysis was performed using a flow cytometer (Navios 10 Colors, Beckman, RRID: SCR_023797, USA) and data were analyzed using ModFit LT software (Verity Software House, Lexington, MA, RRID: SCR_016106, USA).

# **Wound healing assay**

The effect of SNX4 knockdown on the migratory ability of cells (U251 MG, LN229) and the effect of small molecule inhibitors targeting SNX4 on the migratory ability of cells (HA 1800, U251 MG, LN229) were verified by wound healing assay. Cells of the stable SNX4 knockdown U251 MG, LN229 cell lines, and their control cell lines, as well as HA 1800, U251 MG, and LN229 cell lines were inoculated in 6-well cell culture plates at a density of 5×10^5^ cells per well. Once cell confluency reached 90-100%, a straight line was drawn through the monolayer of cells with a vertically held pipette tip to simulate wounding. The cells were then washed with PBS to remove cell debris, then DMEM-free medium was added to the cells in each well. For HA 1800, U251 MG, and LN229 cells, one of the SNX4 small molecule inhibitors, G149 and G431, were added to cells at each of the indicated two different concentrations and the photographs of the scratched wounds were taken at 0 h and 24 h after wounding under an inverted microscope (Axio Scope Al, Carl Zeiss, Germany), and the areas of the scratch wounds were measured using ImageJ software (RRID: SCR_003070) and then the wound closure rates were calculated and plotted.

# **Cell invasion assay**

The effect of SNX4 knockdown on the invasive ability of cells (U87 MG, U251 MG, LN229) and the effect of small molecule inhibitors targeting SNX4 on the invasive ability of cells (U87 MG, U251 MG, LN229) were verified by Transwell assay. Transwell assays were performed using 24-well plates. Each well was placed with a Transwell chamber with an insert with 8 µm pore size and treated with 100 µL of Matrigel (Matrigel:DMEM=1:3) before inoculation of cells in the upper chamber. 5×10^4^ cells/well were suspended in 200 µL free-serum DMEM medium and inoculated in the upper chamber. The lower chamber was supplemented with 800 µL of DMEM containing 10% fetal bovine serum and incubated for 24 h. Among them, U87 MG, U251 MG, and LN229 cells were treated with addition of each of two different concentrations of each of SNX4 small molecule inhibitors, G149 and G431, and then incubated for 24 h and 48 h. Cells were fixed with 4% paraformaldehyde (PFA) for 20 min and stained with 1% crystal violet staining solution for 2 h. The cells were then washed and observed and photographed under an ortho-microtome (Axio Scope Al, Carl Zeiss, Germany). The number of cells invaded through the insert membrane was analyzed using ImageJ software (RRID: SCR_003070).

# **Apoptosis assay**

The effects of SNX4 knockdown on apoptosis of cells (U87 MG, U251 MG, LN229) and the effects of small molecule inhibitors targeting SNX4 on apoptosis of cells (HA 1800, U87 MG, U251 MG, LN229) were verified by flow cytometry. The stable SNX4 knockdown cell lines of U87 MG, U251 MG, and LN229 and their control cell lines, as well as HA 1800, U87 MG, U251 MG, and LN229 cells were inoculated in 6-well cell culture plates at a density of 5×10^5^ cells per well. Among them, HA 1800, U87 MG, U251 MG, and LN229 cells were treated with each of the two different concentrations of each of SNX4 small molecule inhibitors, G149 and G431, and incubated for 24 and 48 h. Cells were treated with Apoptosis Detection Kit and analyzed using a flow cytometer (Navios 10 Colors, Beckman, RRID: SCR_023797, USA).

# **Subcutaneous and orthotopic xenograftment of human GBM cells in nude mouse brain model**

BALB/c nude mice (6-8 weeks, Male, 18-22 g) were provided by Qing Longshan Laboratory Animal Center (Nanjing, China), and the mice were housed in SPF-grade animal rooms with free access to food and water. All animal studies were conducted in accordance with the guidelines of the Chinese Regulations for the Management of Laboratory Animals and approved by the Animal Care and Use Committee (IACUC) of Wannan Medical University. All procedures complied with the regulations of the Institutional Animal Care and Ethics Committee of Wannan Medical University.

Subcutaneous transplantation model: U87 MG stable knockdown cell lines U87 MG-sh1 LUC, U87 MG-sh2 LUC and control cell line U87 MG-shNC LUC (1×10^7^) were subcutaneously injected into BALB/c nude mice (n=3/group), respectively. One week after injection, tumor size and body weight were measured every two days and tumor volume was calculated as length × width^2^/2. Tumors were collected on day 23 post-injection and frozen sections were made for further analysis.

Intracranial orthotopic transplantation model: This model was established according to our previously reported method^1^. Briefly, Cells of U87 MG-sh1 LUC, U87 MG-sh2 LUC stable cell lines and control cell line, U87 MG-shNC LUC were suspended in PBS at a density of 3×10^7^ /ml. Subsequently, mice were anesthetized and the skin was incised along the midline of the head of the mice by approximately 1 cm to expose the skull and fontanelle, and a hole was drilled at a point 1 mm to the right of the anterior fontanel, and then the stereotactic apparatus was adjusted to ensure that the microsyringe was located at the puncture point, and 10 μl of cell suspension was aspirated. The needle was slowly inserted vertically for 4 mm, then retracted for 1 mm, and finally injected slowly at a rate of 2 μl/min and gradually withdrawn after keeping the needle in place for 2 min. One week after tumor formation, the sodium luciferin solution was intraperitoneally injected to each mouse, then the fluorescence intensity resulting from the luciferase-luciferin biochemical reaction in the brains of nude mice (n=5/group) was detected every 5 days using an animal live imaging system (IVIS Lumina Series Ⅲ, PerkinElmer, RRID: SCR_025239, USA). After two weeks, the mice were euthanized and the brains were removed to make frozen sections for subsequent experiments.

# **Assay of the BBB-crossing ability of drug**

To verify whether the inhibitors, G149 and G431, are able to cross the BBB, we separately conjugated G149 and G431 with 4-Chloro-7-nitrobenzoxadiazole (NBD chloride, NBD-Cl) to produce green fluorescence. NBD-Cl itself is a non-fluorescent compound; when it reacts with thiol or amino, the product generates strong fluorescence^2^. G149 or G431 (1 equivalent (eq.), 10 mg) and NaHCO_3_(3 eq., 6.05 mg) were dissolved in 2 ml of deionized water, and then the solution was placed in a reaction flask. NBD-Cl (1 eq., 4.70 mg) that was dissolved in CH_3_CN (2 ml) was added dropwise to the reaction flask. The mixture was incubated for two h, during which time the color of the mixture changed from bright yellow to deep brown that indicated successful displacement of the chlorine by the terminal amine of the alkaloid (G149 or G431) to form the fluorescent NBD adduct. After this, thin layer chromatography (TLC) indicated that all NBD-Cl added to the reaction mixture had been consumed. The reaction mixture was then cooled and filtered through a pad of Celite. The volume was reduced on the rotary evaporator, and the mixture was purified using column chromatography (petroleum ether (PE): ethyl acetate (EA) = 10:1~3:1 elution gradient). The molecular weights of G149-NBD and G431-NBD adducts were detected by high-resolution mass spectrometry to determine whether G149 and G431 were successfully coupled with NBD-Cl.

The nude mice bearing intracranial tumors were randomly divided into two groups (n=3/group). 100 μl of G149-NBD (15 mg/kg) and G431-NBD (15 mg/kg) solution were respectively injected through the tail vein. The fluorescence intensity in the brains of the nude mice was detected at the indicated different time points (0.5 h, 2 h, 6 h, 24 h, 48 h) after drug injection using animal imaging system (IVIS Lumina Series Ⅲ, PerkinElmer, RRID:SCR_025239, USA). Additionally, the fluorescence intensity in the major vital organs (brain, heart, liver, spleen, lungs, kidneys) of the nude mice was measured to analyze whether the drugs crossed the BBB and entered the brain parenchyma.

# **TUNEL** **staining assay**

Paraffin sections of mouse brain tissue were dewaxed, rehydrated, treated with Triton X-100 for 10 min, and washed three times in PBS. TUNEL experiments were performed according to the instructions of the TUNEL kit. Finally, the slides were sealed using an anti-fluorescence quenching sealing solution containing DAPI and scanned by 3D HISTECH digital pathology system (Pannoramic MIDI II, RRID: SCR_024834, Hungary). Apoptotic rates were statistically analyzed using ImageJ software (RRID: SCR_003070).
**HE staining assay**

After dewaxing and rehydration, paraffin sections of mouse brain tissues and the five major organs were stained with hematoxylin staining solution for 8 min, washed with tap water for 5 min, differentiated in differentiation solution for 5 s, and then treated with bluing solution for 1 min. After the hematoxylin staining, the slides with mouse brain tissue sections were stained in Eosin staining solution for 1 min, washed with tap water for 5 min, 70% ethanol for 10 s, 80% ethanol for 10 s, 90% ethanol for 10 s, and anhydrous ethanol for 10 s. The tissue sections were cleared in xylene for 5 min, and then coverslipped with neutral resin sealant. The slides were scanned by 3D HISTECH digital pathology system (Pannoramic MIDI II, RRID: SCR_024834, Hungary).

**References**

1.	Yao Z, et al. Efficiently targeted therapy of glioblastoma xenograft via multifunctional biomimetic nanodrugs. Biomater Res 26, 71 (2022).

2.	Prajapati P, et al. In-vivo microwave-aided spectrofluorimetric characterization of teneligliptin-loaded solid dispersion adsorbate using quality by design approach. Anal Biochem 700, 115785 (2025).

**2. Supplementary Results**

**Fig S1**. **The expression levels of SNX4 in the two public datasets of human gliomas and the relationship of SNX4 with the survival of glioma patients, as well as the expression levels of SNX4 in human GBM cell lines and clinical glioma tissue samples.** (**A**) Analysis of the expression levels of SNX4 in GBM and LGG tissues, compared to normal tissues in the TCGA glioma dataset. (**B**) Analysis of the relative expression differences of SNX4 in the tissues from the WHO grade II, III, and IV glioma patients in the TCGA glioma dataset. (**C**) Correlation analysis of SNX4 expression in GBM patient tissues from the TCGA glioma dataset with the IDH mutation phenotype. (**D**) Correlation analysis of SNX4 expression in GBM patient tissues from the CGGA glioma dataset with the 1p/19q co-deletion phenotype. (**E**) Relative expression analysis of SNX4 in GBM patients of different subtypes (proneural, classical, and mesenchymal) in the CGGA glioma dataset. (**F**) Analysis of the correlation between SNX4 expression and the survival of WHO grade II glioma patients in the CGGA database. (**G**) Analysis of the correlation between SNX4 expression and the survival of WHO grade III glioma patients in the CGGA database. (**H**) The relative expression differences of SNX4 in human astrocyte cell line (HA 1800) and three GBM cell lines (U87 MG, U251 MG, LN229) shown by cytoimmunofluorescence staining (scale bar, 100 μm). (**I**) The quantitative and statistical analyses of the mean fluorescence intensity (MFI) shown in the panel H. (**J**) The relative expression differences of SNX4 in the clinical glioma tissue samples of WHO grade II, III, and IV glioma patients shown by immunofluorescence staining. (**K**) The quantitative and statistical analyses of the fluorescence intensity in the panel J (scale bar, 50 μm). All data were expressed as the mean ±SEM of three independent experiments and statistically analyzed by Student’s t-test for P-values., ** P <0.01; *** P < 0.001; **** P < 0.0001. MFI: mean fluorescence intensity

**Fig S2**. **The effects of SNX4 knockdown on migration, invasion, and apoptosis of GBM cells.** (**A-B**) Wound healing assays showing the effect of SNX4 knockdown on the migration of U251 MG (A) and LN229 (B) cells. (**C**) The quantitative and statistical analyses of the wound closure rates measured in the panels A and B. (**D**) Transwell assays showing the effect of SNX4 knockdown on the invasion of U87 MG, U251 MG, and LN229 cells cultured for 24 h in the indicated groups. (**E**) The quantitative and statistical analyses of the number of invaded cells observed in the panel D. (**F-H**) Flow cytometry showing the effect of SNX4 knockdown on apoptosis of U87 MG (F), U251 MG (G), and LN229 (H). (**I**) The quantitative and statistical analyses of the percentages of the apoptotic cells measured in the panels F-H. (**J-K**) WB showing the effect of SNX4 knockdown on the expression levels of the three key proteins in the apoptosis pathway (J) and the quantitative and statistical analyses of the grayscale values of these key protein bands (K). All data were expressed as the mean ±SEM of three independent experiments and statistically analyzed by Student’s t-test for P-values. Compared with shNC, *P < 0.05; ** P <0.01; *** P < 0.001; **** P < 0.0001.

**Fig S3**. **Investigation of the mechanism of action of SNX4 on GBM.** (**A-B**) The KEGG functional enrichment analysis (A) and (B) showing the number of the DEGs detected by RNA-Seq that is enriched in the signaling pathways (A) and four biological processes (B) in U87 MG-sh1. The cell cycle, apoptosis, and TGFβ signaling pathway are indicated. (**C**) Analysis of the correlation between SNX4 and TGFβ1 in the TCGA glioma dataset. (**D**) Analysis of the correlation between SNX4 and TGFβR2 in the TCGA glioma dataset. (**E-F**) Wound healing assay showing the effect of TGFβ1 agonist, SRI-011381, on migration of stable SNX4 knockdown U251 MG, after U251 MG cells were treated with SRI-011381 for 24 h (E), and the quantitative and statistical analyses of the wound closure rates in the indicated three groups (F). (**G-I**) WB showing the effect of TGFβ1 agonist, SRI-011381, on the expression levels and phosphorylation status of the four key component proteins in the TGFβ1/Smad signaling pathway in stable SNX4 knockdown U87 MG (G) and U251 MG (H) cells, after cells were with SRI-011381 for 24 h, and the quantitative and statistical analyses of the grayscale values of these protein bands detected in the panel H (I). (**J-K**) WB showing the effect of TGFβ1 agonist, SRI-011381, on the three key component proteins in the apoptosis pathway in stable SNX4 knockdown U87 MG and U251 MG cells (J), after cells were treated with SRI-011381, and the quantitative and statistical analyses of the grayscale values of these protein bands detected in the panel J (K). All data were expressed as the mean ± SEM of three independent experiments and statistically analyzed by Student’s t-test for P-values. Compared with shNC, *P < 0.05; ** P <0.01; *** P < 0.001; **** P < 0.0001.

**Fig S4**. **In vivo validation of the effect of SNX4 on the progression of GBM in a subcutaneous and an intracranial orthotopic xenograft GBM tumor model of nude mice.** (**A**) The representative bioluminescence images performed every 5 days of the subcutaneous tumors formed from the subcutaneously injected U87 MG-Luc cells in mice of SNX4 knockdown groups (sh1 and sh2) and control group (shNC) after subcutaneous (top panels) and intracranial orthotopic (bottom panels) injection of U87 MG-Luc cells in mice (n=3 per group). (**B**) Quantitative analysis of the bioluminescence intensity of luciferase--luciferin biochemical reaction occurred in tumor tissues within mice shown in the panel A. (**C**) The representative photomicrographs of IHC staining results showing the protein expression differences of Ki67 in the coronal tissue sections of mouse brain tissue with the intracranial xenograft glioma tissues (encircled in a black oval) in mice of SNX4 knockdown groups (sh1 and sh2) and control group (shNC). (**D**) The representative photomicrographs of the HE results and the IHC staining results showing the protein expression differences of SNX4 and Ki67 in subcutaneous glioma tissues in each indicated group of mice. All data were expressed as the mean ± SEM of three independent experiments and statistically analyzed by Student’s t-test for P-values. Compared with NC, *P < 0.05; ** P <0.01; *** P < 0.001; **** P < 0.0001.

**Fig** **S5**. **Virtual drug screening of candidate SNX4-binding small-molecule compounds and cell-based assay for drug activity screening.** (**A**) The four drug binding pockets (sites) were scored by MOE-site finder based on the four indicated criteria (size, PLB, Hyd, and side) for the likelihood of drug. (**B-E**) The distribution of binding energy score (glide gscore) of each drug in the compound library bound to the site 1 (B), site 2 (C), site 3 (D) and site 4 (E) of SNX4 protein, respectively, as a function of its molecular weight (Mol weight). (**F-H**) CCK8 assay-based measurements of the inhibitory effect of the top 50 compounds (20 μM) with binding affinity for SNX4 protein on the cell vitality of GBM cells U87 MG (F), U251 MG (G), and LN229 (H). All data were expressed as the mean ± SEM of three independent experiments and statistically analyzed by Student’s t-test for P-values. Compared with NC, *P < 0.05; ** P <0.01; *** P < 0.001; **** P < 0.0001. PLB: Protein Ligand Interaction Fingerprints, Hyd: hydrophobicity.

**Fig S6**. **The effects of two small molecule inhibitors G149 and G431 targeting SNX4 on apoptosis, invasion, and migration of GBM cells.** (**A-B**) The representative images of flow cytometry (A) and quantitative and statistical analyses (B), showing the effects of G149 and G431 on apoptosis of HA 1800, U87 MG, U251 MG, and LN229 cells treated with G149 or G431 at the indicated concentrations (0.05 μM and 0.1 μM) and periods of time (24 h and 48 h). (**C-D**) Transwell assay (C) and quantitative and statistical analyses (D) showing the effect of G149 and G431 on invasion of U251 MG and LN229 cells treated with G149 or G431 at the indicated concentrations (0.05 μM and 0.1 μM) for the indicated periods of time (24 h and 48 h). (**E-J**) Wound healing assay (E, G, I) and quantitative and statistical analyses (F, H, J) showing the effect of G149 and G431 on migration of HA 1800 (E-F), U251 MG (G-H), and LN229 (I-J) cells treated with G149 or G431 at the indicated concentrations (0.05 μM and 0.1 μM) for the indicated periods of time (24 h and 48 h). All data were expressed as the mean ± SEM of three independent experiments and statistically analyzed by Student’s t-test for P-values. Compared with NC or 0 μM (vehicle control), *P < 0.05; ** P <0.01; *** P < 0.001; **** P < 0.0001.

**Fig S7**. **The representative histograms of cell cycle detected by flow cytometry and the quantitative and statistical analyses.** (**A-D**) The representative images of flow cytometry (left-side panels, A-D) and quantitative and statistical analyses (right-side panels, A-D), showing the effects of G149 on the cell cycle progression of HA 1800 (A), U87 MG (B), U251 MG (C), and LN229 (D) after the cells treated with G149 at the indicated concentrations (0.05 μM and 0.1 μM) for the indicated periods of time (24 h and 48 h). All data were expressed as the mean ± SEM of three independent experiments and statistically analyzed by Student’s t-test for P-values. Compared with 0 μM (vehicle control), *P < 0.05; ** P <0.01; *** P < 0.001; **** P < 0.0001.

**Fig S8**. **Detection of the ability of G149 to cross the BBB.** (**A**) Chemical conjugation equation of G149 with NBD-Cl. The conjugation occurs at the groups encircled in red ovals. (**B**) High-resolution mass spectrometry detection of the molecular weight of G149-NBD conjugate as shown in the red box. (**C**) The representative images showing the co-localization (shown as the yellow color) of G149-NBD with SNX4 protein in GBM cells (U87 MG, U251 MG, and LN229) detected by cytoimmunofluorescence. (**D**) The representative intracranial fluorescence images of the whole body of mouse detected by animal live imaging at the different time points (0.5 h, 2 h, 6 h, and 24 h) after tail vein injection of G149-NBD in intracranial orthotopic GBM mouse model. (**E**) The quantitative and statistical analyses of the intracranial fluorescence intensity in mice in the panel D. (**F**) The representative fluorescence images of the five major organs (heart, liver, spleen, lung, kidney) removed from euthanized mice. (**G**) The representative intracranial fluorescence images of the whole body of mouse detected by animal live imaging at the different time points (0.5 h, 2 h, 6 h, and 24 h) after tail vein injection of G149-NBD in normal mouse. (**H**) The quantitative and statistical analyses of the intracranial fluorescence intensity in mice in the panel G. All data were expressed as the mean ± SEM of three independent experiments and statistically analyzed by Student’s t-test for P-values. Compared with 0.5 h, *P < 0.05; ** P <0.01; *** P < 0.001; **** P < 0.0001.

**Fig S9**. **Detection of the ability of G431 to cross the BBB.** (**A**) Chemical conjugation equation of G431 with NBD-Cl. The conjugation occurs at the groups encircled in red ovals. (**B**) High-resolution mass spectrometry detection of the molecular weight of G431-NBD conjugate as shown in the red box. (**C**) The representative images showing the co-localization (shown as the yellow color) of G431-NBD with SNX4 protein in GBM cells (U87 MG, U251 MG, and LN229) detected by cytoimmunofluorescence. (**D**) The representative intracranial fluorescence images of the whole body of mouse detected by animal live imaging at the different time points (0.5 h, 2 h, 6 h, 24 h and 48 h) after tail vein injection of G431-NBD in intracranial orthotopic GBM mouse model. (**E**) The quantitative and statistical analyses of the intracranial fluorescence intensity in mice in the panel D. (**F**) The representative fluorescence images of the five major organs (heart, liver, spleen, lung, kidney) removed from euthanized mice. (**G**) The representative intracranial fluorescence images of the whole body of mouse detected by animal live imaging at the different time points (0.5 h, 2 h, 6 h, 24 h and 48 h) after tail vein injection of G431-NBD in normal mouse. (**H**) The quantitative and statistical analyses of the intracranial fluorescence intensity in mice in the panel G. All data were expressed as the mean ± SEM of three independent experiments and statistically analyzed by Student’s t-test for P-values. Compared with 0.5 h, *P < 0.05; ** P <0.01; *** P < 0.001; **** P < 0.0001.

**Fig S10**. **The representative bioluminescence images of the intracranial orthotopic GBM tumors in nude mice treated with two inhibitors.** (**A-C**) The representative bioluminescence images of intracranial orthotopic GBM tumors detected by animal live imaging on the fourth and eighth days after treatment in mice from control (NC), G149-, and G431-treated groups (n=5).

**Fig S11**. **The representative photomicrographs of HE staining and IHC staining of the coronal sections of mouse brain with orthotopic GBM tumors in mice treated with two inhibitors.** The photomicrographs of HE staining (top panels) and IHC staining (bottom panels) of the coronal sections of mouse brain with orthotopic GBM tumors (encircled by red or black ovals) show the protein expression differences of SNX4 and Ki67 in intracranial tumor tissues of mice in each group.

**Fig S12**. **The representative photomicrographs of HE staining of the five major organs from the nude mice treated two inhibitors.** The photomicrographs of HE staining show no discernible toxicity of two inhibitors to the five major organs (heart, liver, spleen, lung, kidney) in mice in the different groups (scale bar, 50 μm).

**Fig S13**. **RNA-Seq analysis for the elucidation of the mechanism of action of two small molecule inhibitors G149 and G431 targeting SNX4 on GBM.** (**A**) KEGG functional enrichment analysis of RNA-Seq data from U87 MG cells treated with G149. (**B-C**) GO functional enrichment analysis of RNA-Seq data from U87 MG cells treated with G149. (D) KEGG functional enrichment analysis of RNA-Seq data from U87 MG cells treated with G431. (**E-F**) GO functional enrichment analysis of RNA-Seq data from U87 MG cells treated with G431. (**G**) Venn diagram of the combined analysis of RNA-Seq results from SNX4 knockdown U87 MG cells and U87 MG cells treated with G149 and G431.

**Fig S14**. **Fluorescent staining detection of the effects of G149 and G431 on EMT and apoptosis of intracranial GBM tissue in mice.** (**A-B**) The representative photomicrographs of the immunofluorescence staining (A) showing the expression levels of EMT markers (E-cadherin and Vimentin) in the coronal sections of mouse brain with orthotopic GBM tumor (marked by red ovals) in the indicated groups and the statistical analysis of immunofluorescence staining results (B). E-cadherin: green; Vimentin: red; DAPI: blue. (**C-D**) The representative photomicrographs of TUNEL staining (C) of the coronal sections of mouse brain with orthotopic GBM tumor (marked by red ovals) in the indicated groups and the statistical analysis of the TUNEL staining results (D). All data were expressed as the mean ± SEM of three independent experiments and statistically analyzed by Student’s t-test for P-values. Compared with NC, *P < 0.05; ** P <0.01; *** P < 0.001; **** P < 0.0001.
